# Exploring the changes in functional connectivity of the limbic system in Patients with amnestic mild cognitive impairment treated by acupuncture based on fMRI

**DOI:** 10.3389/fneur.2025.1506367

**Published:** 2025-06-13

**Authors:** Han Yingmei, Li Yijie, Zhang Heng, Feng Ze, Li Weiqing, Zhang Hanxi, Yang Ming, Chu Bingyuan, Wang Feng

**Affiliations:** ^1^Graduate School of Heilongjiang University of Chinese Medicine, Harbin, China; ^2^Division of CT and MRI, First Affiliated Hospital, Heilongjiang University of Chinese Medicine, Harbin, China

**Keywords:** limbic system, functional connectivity, acupuncture, functional magnetic resonance imaging, amnestic mild cognitive impairment

## Abstract

**Objective:**

Brain functional connectivity (FC) of Limbic system plays an important role in maintaining the normal cognitive state. We conduct an investigation of the FC of limbic system networks in amnestic mild cognitive impairment (aMCI) and speculate on the brain effect mechanism of acupuncture therapy based on resting - state Functional Magnetic Resonance Imaging (rs - fMRI).

**Method:**

50 patients with aMCI and 41 healthy participants (HC group) from the First Affiliated Hospital of Heilongjiang University of Chinese Medicine in Harbin City, Heilongjiang Province, China, were recruited.rs-fMRI data of all participants were collected. Among them, 35 aMCI participants (true-acupoint group) were treated with the Yuanluo Tongjing acupuncture method for two courses of treatment (once a day, needling every 10 min, retaining the needles for 40 min, 6 days of treatment + 1 day of rest, 4 weeks as one course, and starting the second course after an interval of 2 weeks). 15 aMCI participants (sham-acupoint group) received sham acupoint acupuncture intervention, and the specific intervention details were the same as those of the true-acupoint group. After treatment, rs-fMRI data of aMCI subjects were collected again. Thirty seed points of the limbic system were selected based on the Anatomical Automatic Labeling (AAL) template, and the Statistical Parametric Mapping (SPM) software was used for statistical analysis of FC indices between and within groups.

**Result:**

(1) Compared with the HC group, there were significant differences in the FC between Seed14 of the true-acupoint group before acupuncture intervention and multiple brain regions (enhanced with Seed 7 and weakened with Seed 15). There were differences in the FC of Seed4, Seed29, and Seed30 in the sham-acupoint group, indicating that there were baseline differences among aMCI patient groups. (2) After acupuncture in the true-acupoint group, the FC between multiple seed points and brain regions decreased, while the differences before and after the intervention in the sham-acupoint group mostly did not pass the Family-Wise Error (FEW) correction. (3) Compared with the HC group, the FC of seed points in both the true-acupoint group and the sham-acupoint group mainly decreased after acupuncture. The true-acupoint group involved a wider range of brain regions (the middle frontal gyrus, the left medial superior frontal gyrus, the middle part of the left cingulate gyrus and the gyri surrounding its lateral side, the gyri below the bilateral parietal bones except the supramarginal gyrus and the angular gyrus, the precuneus, etc.). (4) The FC between Seed14 and the left superior frontal gyrus medialis (Seed7), as well as the right caudate nucleus of the true-acupoint group was enhanced before acupuncture and decreased after acupuncture, which may serve as an observational indicators for the intervention of aMCI by acupuncture at acupoints. (5) The Montreal Cognitive Assessment (MoCA) score is more representative in characterizing the abnormal FC between brain regions in aMCI patients.

**Conclusion:**

The cerebral effect mechanism of acupuncture at acupoints for aMCI is more complex. It can regulate the functional connections within the limbic system and between the limbic system and other brain regions, mainly manifested as a decrease. Among them, the FC among Seed17-Parietal_Inf_L, Seed25-Frontal_Mid_L, and See25-Frontal_Sup_Medial_L has become a statistically significant detection index.

## Graphical abstract

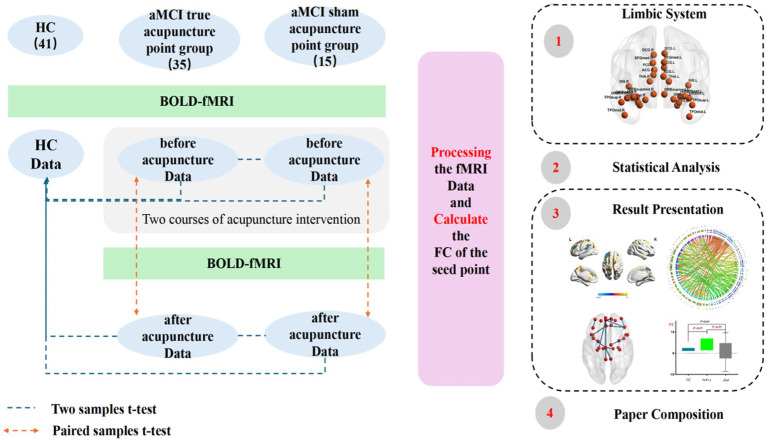

## Highlights

- This experiment uses the Yuanluo Tongjing acupuncture method of TCM to treat patients with aMCI. This acupuncture method is based on the overall concept and dialectical treatment of TCM, and multiple acupoints cooperate with each other, which is demonstrated as an effective method to treat cognitive dysfunction.
- The limbic system network is relatively complex in structure and function, and the main brain regions involved are the main nodes of multiple brain networks, such as the Default Mode Network (DMN). Moreover, there are relatively few studies on limbic system network, so from the perspective of edge network, we can have a more comprehensive understanding of the large-scale brain network in aMCI patients.
- With the help of functional MRI imaging technology, the brain neurons are detected to explore the functional connectivity changes of limbic system, which provides a visual analysis method.

## 1 Introduction

Alzheimer’s disease (AD) is an age-related neurodegenerative disease in which patients mainly manifest as a decline in memory, executive ability, spatial ability, language, emotion and other systems, which is a continuous development process (1, 2). Mild cognitive impairment (MCI) is a transitional stage between healthy older individuals and AD (3). Not all MCI patients progress to AD, as MCI comprises several subtypes. Among these, amnestic MCI (aMCI), characterized primarily by memory impairment, is the subtype most likely to develop into AD (4). Early identification of this subtype and the exploration of effective interventions can significantly improve clinical outcomes (5). In Western medicine (6, 7), the treatment of AD primarily involves medications such as memantine, donepezil, galantamine, and other neurotransmitter system medications, but these cannot effectively halt the progression of the disease. In recent years, emerging machine-based stimulation therapies such as repetitive transcranial magnetic stimulation (rTMS) (8–10) and computerized cognitive training (11, 12) have been used to evaluate the efficacy of treatment for MCI and AD by monitoring improvements in network topology properties or functional connectivity between brain regions. Inspired by this, an increasing number of researchers have explored the efficacy of acupuncture in treating aMCI. A multitude of studies have subsequently demonstrated that acupuncture yields remarkable therapeutic outcomes for this condition. (13–15). Functional magnetic resonance imaging (fMRI) technology has made remarkable contributions to the research of imaging biomarkers in aMCI and AD. At the same time, it has also become an important tool for the visual presentation of both the therapeutic effects of acupuncture treatment for these diseases and its regulatory mechanisms.

According to the existing literature, in the research of the keyword co-occurrence analysis diagram of acupuncture treatment for MCI, keywords such as Alzheimer’s disease (AD), acupuncture, electroacupuncture, brain, activation, and Baihui (GV20) occupy a major position, and the number of relevant English papers published increases year by year (6, 7, 16). Further research indicates that acupuncture treatment can significantly enhance the overall cognitive function of MCI patients. Among them, commonly used acupoints include Baihui (GV20), Sishencong (EX-HN1), and Shenting (GV24) (17). Meanwhile, in the meta-analysis of magnetic resonance imaging-related literature on acupuncture treatment for MCI, it has been found that regardless of whether the experimental design is in the rest state or the task state, the acupuncture therapy indeed has a regulatory effect on the brain regions of MCI patients (18). An in-depth exploration of its mechanism reveals that the brain effect of acupuncture regulation mainly occurs in the Default Mode Network (DMN), Central Executive Network (CEN), and Salience Network (SN), especially in the cingulate cortex, hippocampus, and prefrontal cortex (17, 19). It is noteworthy that compared with the sham acupoint group, acupuncture at real acupoints can lead to an increase in brain activity with a wider range of changes. The mainly activated brain regions include cognitive-related regions (inferior frontal gyrus, middle temporal gyrus, supramarginal gyrus, etc.), sensorimotor-related regions (superior parietal gyrus), basal ganglia (globus pallidus), cerebellum, limbic system, and advanced cognitive regions (20). In addition, some researchers have tested the effects of two acupuncture methods, deep acupuncture (acupuncture depth of 1–2 centimeters) and shallow acupuncture (acupuncture depth of 1–2 millimeters), on subjects with AD and MCI in the early stage. It has been found that the two acupuncture methods present a heterogeneous regulation pattern, and the clinical effect of deep muscle acupuncture is more significant (21).

In the proportion of scanning techniques for exploring the neuronal activity, changes in brain function, changes in brain structure, metabolic ratios, and hemodynamic responses in the human brain of MCI patients induced by acupuncture, functional magnetic resonance imaging (fMRI) accounts for the largest proportion, which is 74% (17, 19). Research has found that the abnormal activity in multiple specific brain regions may be a manifestation of impaired central nervous system function in patients with aMCI. During the aMCI stage, imaging techniques have detected abnormalities in the structure, function, neuronal activity, and metabolism of brain regions such as the hippocampus and cingulate gyrus (22–24), as well as abnormal functional connectivity across multiple functional networks (25). Additionally, asymmetrical changes in the anatomical structure of nuclei such as the amygdala and nucleus accumbens have been observed (26). The brain regions mentioned above constitute a more complex neural network system in the human brain, known as the limbic system. The limbic system, due to its intricately interconnected synapses, does not have clearly defined structural boundaries in its constituent regions. It primarily serves as the emotional control system of the human brain, and is closely related to cognitive and behavioral abilities. Morphologically, it is divided into two major categories. The first belongs to limbic cortex, is located between the cortex and subcortical structures, mainly including the hippocampus, parahippocampal gyrus, cingulate gyrus, orbitofrontal cortex, and insular cortex. And the second comprises subcortical structures, include the amygdala, nucleus accumbens, and medial nuclei of the thalamus, among others (27). The limbic network constructed via the limbic system is mainly composed of the temporal poles and the regions of the orbitofrontal cortex (28).

The structural topology of the limbic system in MCI patients has been disrupted, mainly involving brain regions such as the hippocampus, anterior cingulate gyrus, and posterior cingulate gyrus (29). Among them, the amygdala is the core hub of emotions, behaviors, and memory in the limbic system, and it also regulates the body’s responses to stress, attention, and sexual instincts (30). Additionally, the volume of the amygdala is closely related to an individual’s arithmetic and financial abilities (31). Studies have also found that metacognitive avoidance strategies are correlated with the volume of the bilateral amygdala at baseline and with the volume of the bilateral parahippocampus during follow-up, suggesting that these brain regions can be used as detection indicators for metacognitive knowledge deficits in aMCI patients (32). Research on the radiomic features of the amygdala has speculated that it may serve as an early biomarker for detecting changes in the microstructural tissues of the brain during the evolution from aMCI to AD (33). Other studies have shown that there is insufficient cerebral perfusion in the limbic network of aMCI patients, which supports using the cerebral perfusion of the limbic network as an effective biomarker for the conversion of aMCI to AD (34). Abnormalities in the structure, function, metabolism, and perfusion of the limbic system can easily affect patients’ emotional regulation, social interaction, and other behavioral manifestations, posing potential risks to both patients themselves and caregivers (35). Therefore, in-depth research on the limbic network is of great significance for exploring the memory function of the brain and elucidating the pathogenesis of mental diseases (36, 37). However, currently, the research efforts on the limbic system in the academic community are far less than those on popular research areas such as the default mode network, executive control network, and salience network.

Therefore, this study takes aMCI patients as the main research subjects and uses the acupuncture method of the Yuanluo Tongjing Acupuncture Technique to treat aMCI patients (38), and observes the changes in the internal functional connections of the limbic system. Based on the research experience of predecessors and the Anatomical Automatic Labeling (AAL) template of the human brain, we selected the seed points of the LN, including the superior frontal gyrus orbital part, middle frontal gyrus orbital part, inferior frontal gyrus orbital part, superior frontal gyrus medialis, superior frontal gyrus orbital part medialis, insula, anterior cingulate gyrus and the gyri surrounding its lateral side, middle cingulate gyrus and the gyri surrounding its lateral side, posterior cingulate gyrus and the gyri surrounding its lateral side, hippocampus, parahippocampal gyrus, insula, amygdala, thalamus, temporal pole of the superior temporal gyrus, and temporal pole of the middle temporal gyrus.

## 2 Materials and methods

### 2.1 Participants

Recruit patients who were first diagnosed with aMCI and visited the Acupuncture Department outpatient clinic at the First Affiliated Hospital of Heilongjiang University of Chinese Medicine (hereinafter referred to as “our hospital”) between April 2022 and April 2025.

Previous studies have demonstrated that in imaging research, a larger sample size is associated with higher credibility of the obtained results (39). However, in practical experiments, the magnetic resonance sequence scanning incurs high costs, and there are certain difficulties in the subjects’ cooperation. As a result, the sample size in most current neuroimaging studies is relatively small, generally controlled at around 20 cases. Additionally, some researchers hold the view that a sample size of 12 cases is sufficient to meet the requirements for reliable statistical analysis (40). “Acupuncture Imaging” states that “when ethical and experimental conditions permit, the sample size of a single group should preferably reach more than 20 cases.” Based on this, a total of 91 subjects were ultimately included in this study for statistical analysis, among which there were 41 subjects in the healthy control group (HC) (including 17 males and 24 females, aged 55–75 years) and 50 subjects in the aMCI group (21 males and 29 females, aged 55–75 years). For the aMCI subjects, a non-randomized controlled method was employed for grouping, with 35 subjects in the true-acupoint group and 15 subjects in the sham-acupoint group. The participants voluntarily joined this trial, understood and signed the informed consent form, and obtained approval from the Ethics Committee of the First Affiliated Hospital of Heilongjiang University of Chinese Medicine (Ethical number: HZYLLKY202001101, 2020.08.27).

#### 2.1.1 Inclusion criteria for the aMCI

① Aged between 55 and 75 years old; ② Selection of individuals without bad habits, such as non-smoking and non-alcoholism; ③ Self-reported or reported by others to have mild memory problems; ④ The scoring criteria of the Mini-Mental State Examination (MMSE) scale: for the illiterate group, the score is ≥ 17 points; for the primary school education group, the score is ≥ 20 points; for the group with junior high school education or above, the score is ≥ 24 points. In the tests of memory or other cognitive domains, the performance is lower than that of peers but higher than 1.5 standard deviations below the standard score, and the score of the Clinical Dementia Rating (CDR) scale is 0.5 points; ⑤ The total score of the Montreal Cognitive Assessment (MoCA) scale is ≤ 26 points, and other diseases that can cause cognitive impairment, such as brain trauma, stroke, Parkinson’s disease, and hypothyroidism, are excluded; ⑥ The results of cranial MRI examination are normal, and the skin at the acupuncture site is intact without scars; ⑦ Before the experiment, the individuals have not received acupuncture treatment in the past two months and have no physical discomfort.

#### 2.1.2 Inclusion criteria for the HC group

① No obvious behavioral and language disorders; ② Normal cognitive function assessment; ③ Right-handed; ④ No neurological diseases, etc.

#### 2.1.3 Exclusion criteria

① Obvious dementia; ② Having impairments such as visual, auditory, or aphasic disorders, or other severe cognitive impairments, such as Alzheimer’s disease, Parkinson’s disease, etc.; ③ Suffering from major mental illnesses or severe visceral dysfunctional diseases of the heart, liver, kidneys, etc.; ④ Long-term use of drugs that affect cognitive abilities, such as benzodiazepines, anticholinergic drugs, etc.; or those who have taken sedative drugs within the last month before the trial; or having diseases that may affect cognitive performance, such as epilepsy, brain tumors, severe head trauma, etc.; ⑤ Drug abuse or alcoholism; ⑥ Having a pacemaker or metal fragments in the body that affect magnetic resonance imaging (MRI) scanning; ⑦ Having psychological diseases such as claustrophobia and being unable to undergo MRI scanning; ⑧ Congenital cranial malformation; ⑨ Color blindness, etc.

#### 2.1.4 Acupuncture methods

**Figure 1:**
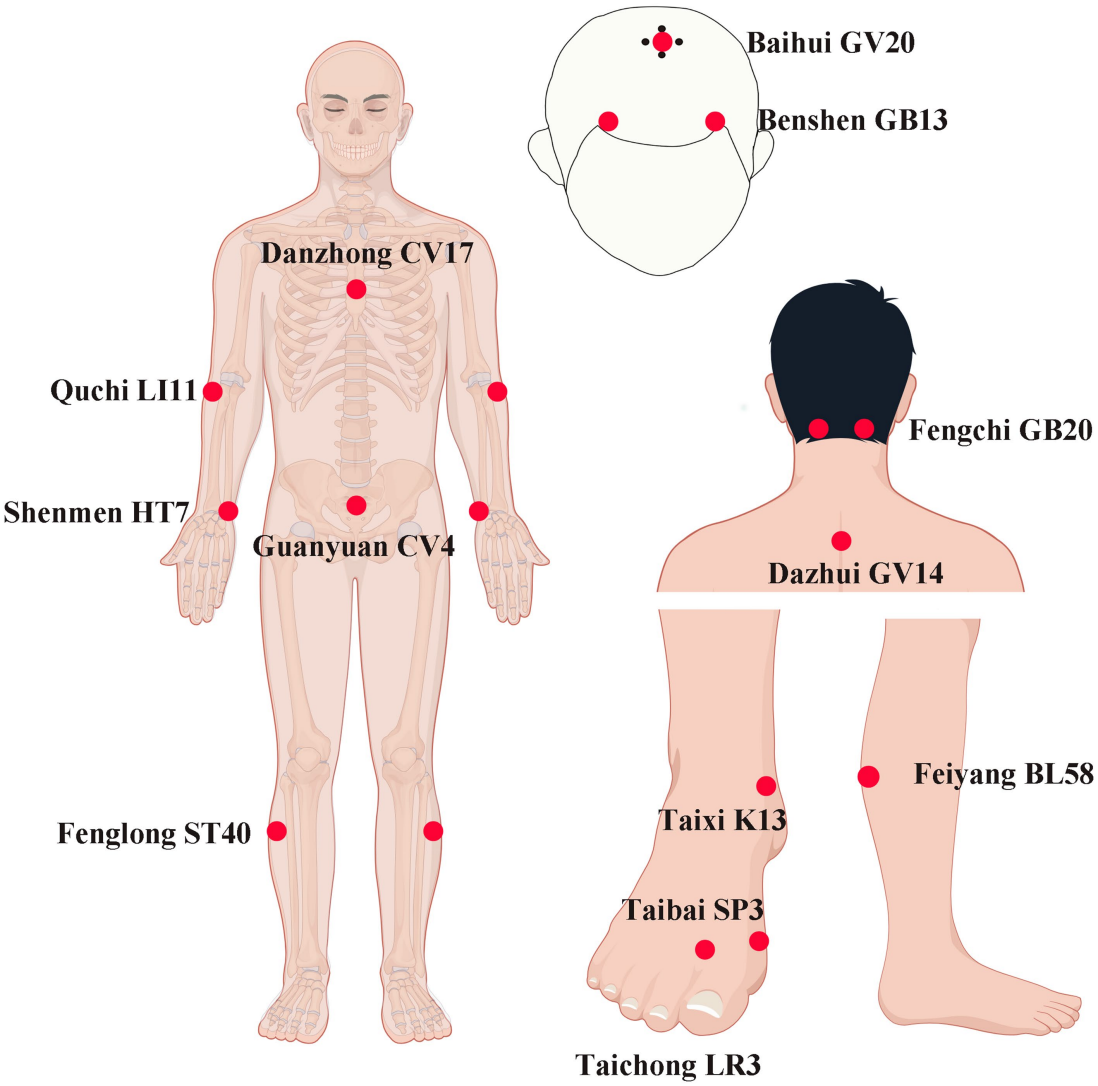
The acupuncture points for the Yuanluo Tongjing method in this study. LI11 (Quchi): on the lateral aspect of the elbow, at the midpoint of the line connecting LU5 with the lateral epicondyle of the humerus. CV17 (Danzhong): in the anterior thoracic region, at the same level as the fourth intercostal space, on the anterior median line. HT7 (Shenmen): on the anteromedial aspect of the wrist, radial to the flexor carpi ulnaris tendon, on the palmar wrist crease. CV4 (Guanyuan): on the lower abdomen, 3 B-cun inferior to the centre of the umbilicus, on the anterior median line. ST40 (Fenglong): on the anterolateral aspect of the leg, lateral border of the tibialis anterior muscle, 8 B-cun superior to the prominence of the lateral malleolus. K13 (Taixi): On the posteromedial aspect of the ankle, in the depression between the prominence of the medial malleolus and the calcaneal tendon. SP3 (Taibi): on the medial aspect of the foot, in the depression proximal to the first metatarsophalangeal joint, at the border between the red and white flesh. LR3 (Taichong): On the dorsum of the foot, between the first and second metatarsal bones, in the depression distal to the junction of the bases of the two bones, over the dorsalis pedis artery. BL58(Feiyang): On the posterolateral aspect of the leg, between the inferior border of the lateral head of the gastrocnemius muscle and the calcaneal tendon, at the same level as 7 B-cun proximal to BL60. GV14 (Dazhui): in the posterior region of the neck, in the depression inferior to the spinous process of the seventh cervical vertebra (C7), on the posterior median line. GB20 (Fengchi): ferior to the occipital bone, in the depression between the origins of sternocleidomastoid and the trapezius muscles. GV20 (Baihui): on the head, 5 B-cun superior to the anterior hairline, on the anterior median line. GB13 (Benshen): on the head, 0.5 B-cun superior to the anterior hairline, 3 B-cun lateral to the anterior median line. By Figdraw.

Ture-acupoint Group: Using the Yuanluo Tongjing acupuncture method, which is based on the theory of host-guest Yuanluo point pairing, this technique has been widely applied in the field of cognitive impairment with significant efficacy. The diagram for point selection according to the WHO Standard Acupuncture Locations is shown in Figure 1. Bilateral Shenmen (HT7), Taixi (KI3), Feiyang (BL58), Taibai (SP3), Fenglong (ST40), Fengchi (GB20), Quchi (LI11), Taichong (LR3) as well as Baihui (GV20), Dazhui (GV14), Danzhong (CV17), and Guanyuan (CV4) acupoints were selected. The patient was seated, and the corresponding acupoint areas of the subject were routinely disinfected. A disposable Huatuo-brand stainless steel flat-handle acupuncture needle (0.30 × 40 mm) produced by Jiangsu Medical Supplies Factory Co., Ltd. was used for acupuncture. The needle was quickly inserted vertically to a depth of approximately 0.5 cun. The reinforcing-reducing manipulation was applied, and the needle twirling was carried out. The duration of needle twirling was 60 s. The twirling angle was controlled within the range of 180° ± 20°, and the frequency was 60–90 times per minute. During the needle twirling process, the subject would experience the deqi sensations such as soreness, numbness, distension, and heaviness. After achieving deqi, the needle was retained for 40 min. The treatment was administered once a day, needling every 10 min, retaining the needles for 40 min, 6 days of treatment + 1 day of rest, 4 weeks as one course, and starting the second course after an interval of 2 weeks.

Sham-acupoint Group: The acupoints for acupuncture in this group are all non-meridian and non-acupoints. They are located 0.5 cun away from the actual acupoints to be needled, bypassing the acupoints. The acupuncture intervention measures and the protocol are all consistent with those of the true-acupoint group.

During this process, the acupuncture is carried out by an acupuncturist who has more than 10 years of professional experience.

### 2.2 Neuropsychological scale scoring

We evaluated the clinical cognitive status of each participant before and after the acupuncture intervention. The Mini-Mental State Examination (MMSE) and the Montreal Cognitive Assessment (MoCA) were sequentially administered to assess multiple cognitive domains of the participants, such as memory, attention, computational ability, recall ability, language ability, and executive function. Taking into account the learning effects of the subjects and avoiding potential risks, we decided to conduct the scale evaluations twice for both the HC group and the aMCI group, which were completed within one week after the acupuncture intervention course and at the time of enrollment. During the evaluation process, the assessments were carried out by the same researcher (with experience in using the scales) in the same testing environment.

### 2.3 Preparation before the experiment

Most of the subjects have not experienced acupuncture and cranial magnetic resonance examination, and their psychological state may affect the accuracy of the experiment. Before the experiment, the acupoint information was concealed, and the process was informed to relieve their anxiety. Meanwhile, before the experiment, the subjects were allowed to eat an appropriate amount of caffeine-free food within 2 h, until they were about 70% full; intense exercise was avoided within 30 min; they were required to fill in the personal information form and sign the informed consent form. During the experiment: ① The subjects were required to remove metal objects and items that could interfere with the magnetic field and change into the experimental clothing; ② Earplugs and eye masks were worn after entering the magnetic field; ③ The subjects lay supine on the scanning table, placed their heads in the coil, and remained still. If the subjects wanted to terminate the experiment, they could wave to indicate.

### 2.4 Image acquisition

The experiment used a Philips Ingenia 3.0 T fully digital MRI scanner with a gradient field strength of 40 mT/m, a 16-channel parallel head coil (SENSE-NV-160), and an 80 MHz high-frequency analog-to-digital converter for each channel, eliminating the need for analog filtering through direct digital sampling. The gradient switching rate was 200 mT/m/ms. Functional imaging was acquired using a single-shot fast echo planar imaging (Field Echo–Echo Planar Imaging, EPI) sequence. The functional image scanning parameters were as follows: TR = 2000 ms, TE = 30 ms, FOV = 220 mm × 220 mm × 143 mm, flip angle = 90°, matrix = 64 × 64, number of slices = 36, slice thickness = 3 mm, slice gap = 1 mm, and total scan duration = 6min6s. Acquisition was performed at a total of 180 timepoints. The scan covers the entire brain. The HC group had a single BOLD-fMRI scan upon enrollment. The true - and sham - acupoint groups had two BOLD-fMRI scans: one at enrollment and the other after two acupuncture treatment courses.

### 2.5 Image preprocessing

The collected rs-fMRI image data were preprocessed using the Data Processing Assistant for Resting-State fMRI (DPARSFA) toolkit software based on the MATLAB platform. The main preprocessing steps included: converting DICOM format to NIFIT format; removing the first 10 time points; time correction; head motion correction; spatial registration, noise regression, band-pass filtering, and spatial smoothing, etc. VBM methods were applied to analyze brain regions with significant differences, and changes in the strength of functional connectivity between brain regions were subsequently assessed. The steps for calculating FC metrics are as follows: ① Extract the time series of the ROIs and compute the average time series for each ROI. ② Use the average time signal of the voxels within each ROI as the seed point signal and analyze the Pearson correlation coefficient between this seed point and the time series of each voxel across the whole brain. ③ Apply Fisher’s Z transformation to convert the correlation coefficients into Z-scores for normalization, resulting in a brain functional connectivity image for each subject.

### 2.6 Statistical processing and analysis methods

Image data processing used Statistical Parametric Mapping software (SPM12, http://www.fil.ion.ucl.ac.uk/spm) for group analysis of seed point FC metrics. A two-sample t-test was performed to compare FC values between the control group and the pre-acupuncture aMCI group, while a paired sample t-test was used to compare pre- and post-acupuncture FC values within the aMCI group. Statistical thresholds were set at voxel-level *p* = 0.001 (uncorrected) and cluster-level *p* < 0.05 (FWEc corrected). Results were presented using software such as xjView and BrainNet Viewer.

The statistical software SPSS23.0 was used to analyze the clinical data of the subjects, including general information (age, gender, years of education) and scores of neuropsychological scales. The data were expressed as mean ± standard deviation (SD). Chi-square test, two-sample t-test, and Mann–Whitney U test were used for intergroup comparisons. Paired sample t-test and Wilcoxon signed-rank test were employed for intragroup comparisons. A *p* value less than 0.05 was considered statistically significant. The results were presented using Prism software.

## 3 Results

### 3.1 General data

There were no significant statistical differences between the aMCI and HC groups in demographics (age, gender, years of education) (*p* > 0.05). However, there were significant statistical differences in neuropsychological scores (MMSE, MoCA) (*p* < 0.05). See Table 1 for details.

**Table 1:** Comparison of demographic and neuropsychological scores between the aMCI and HC groups.

	HC (41)	aMCI (35) (before treatment)	aMCI (15) (before treatment)	*p* values
Demography				
Age	63.24 ± 5.37	64.37 ± 5.40	63.47 ± 10.09	0.735
Sex (male: female)	18:23	16:19	8:7	0.979
Education level(years)	11.80 ± 2.50	11.51 ± 2.65	13.07 ± 4.70	0.244
Neuropsychological score				
MMSE (points)	28.22 ± 0.96	24.06 ± 1.41	25.00 ± 1.81	0.000
MoCA (points)	27.34 ± 0.96	22.31 ± 2.89	22.80 ± 3.12	0.000

*p* < 0.05 indicates statistical significance. Categorical variables (gender) were analyzed using the *χ*^2^ test; normally distributed data were analyzed using an independent sample *t*-test (mean ± standard deviation); non-normally distributed data were analyzed using the Mann–Whitney *U* test [median (interquartile range)]. HC, healthy controls; aMCI, amnestic mild cognitive impairment; MMSE, Mini-Mental State Examination; MoCA, montreal cognitive assessment.

Paired - sample *t* - tests were conducted to analyze the scores of neuropsychological scales at two time points for three groups of subjects. The results showed that there were no significant improvements in the MMSE and MoCA scores for the HC group and the sham - acupoint group. In contrast, the MMSE and MoCA scores of the true - acupoint group increased significantly. The detailed data are presented in Table 2.

**Table 2:** Comparison of the scores of the neuropsychological scale at two times among the three groups.

Group	*N*	Time	MMSE	MoCA
HC	41	First	28.22 ± 0.96	27.34 ± 0.96
		Second	28.29 ± 0.93	27.43 ± 0.98
		*P* value	0.083	0.103
aMCI(true-acupoint)	35	First	24.06 ± 1.41	22.31 ± 2.89
		Second	26.17 ± 0.82	24.46 ± 1.74
		*P* value	0.000*	0.000*
aMCI(sham-acupoint)	15	First	25.00 ± 1.81	22.80 ± 3.12
		Second	25.40 ± 1.40	23.07 ± 3.01
		P value	0.054	0.104

*Indicates *p* < 0.05. For within - group score data showing a normal distribution, paired - sample *t* - tests were used, and the results are presented as mean ± standard deviation. A *P* - value < 0.05 was considered statistically significant.

### 3.2 Before acupuncture, functional connectivity changes of seed points among the true acupoint group, sham acupoint group, and HC group

Select 30 seed points according to the main brain regions constituting the limbic system and the limbic network, which are, respectively, the bilateral superior frontal gyrus of the orbital part (Seed1, Seed2), middle frontal gyrus of the orbital part (Seed3, Seed4), inferior frontal gyrus of the orbital part (Seed5, Seed6), medial superior frontal gyrus (Seed7, Seed8), medial superior frontal gyrus of the orbital part (Seed9, Seed10), insula (Seed11, Seed12), the anterior cingulate gyrus and the gyri surrounding its lateral side (Seed13, Seed14), the middle cingulate gyrus and the gyri surrounding its lateral side (Seed15, Seed16), the posterior cingulate gyrus and the gyri surrounding its lateral side (Seed17, Seed18), hippocampus (Seed19, Seed20), parahippocampal gyrus (Seed21, Seed22), amygdala (Seed23, Seed24), thalamus (Seed25, Seed26), temporal pole of the superior temporal gyrus (Seed27, Seed28), and temporal pole of the middle temporal gyrus (Seed29, Seed30).

**Figure 2:**
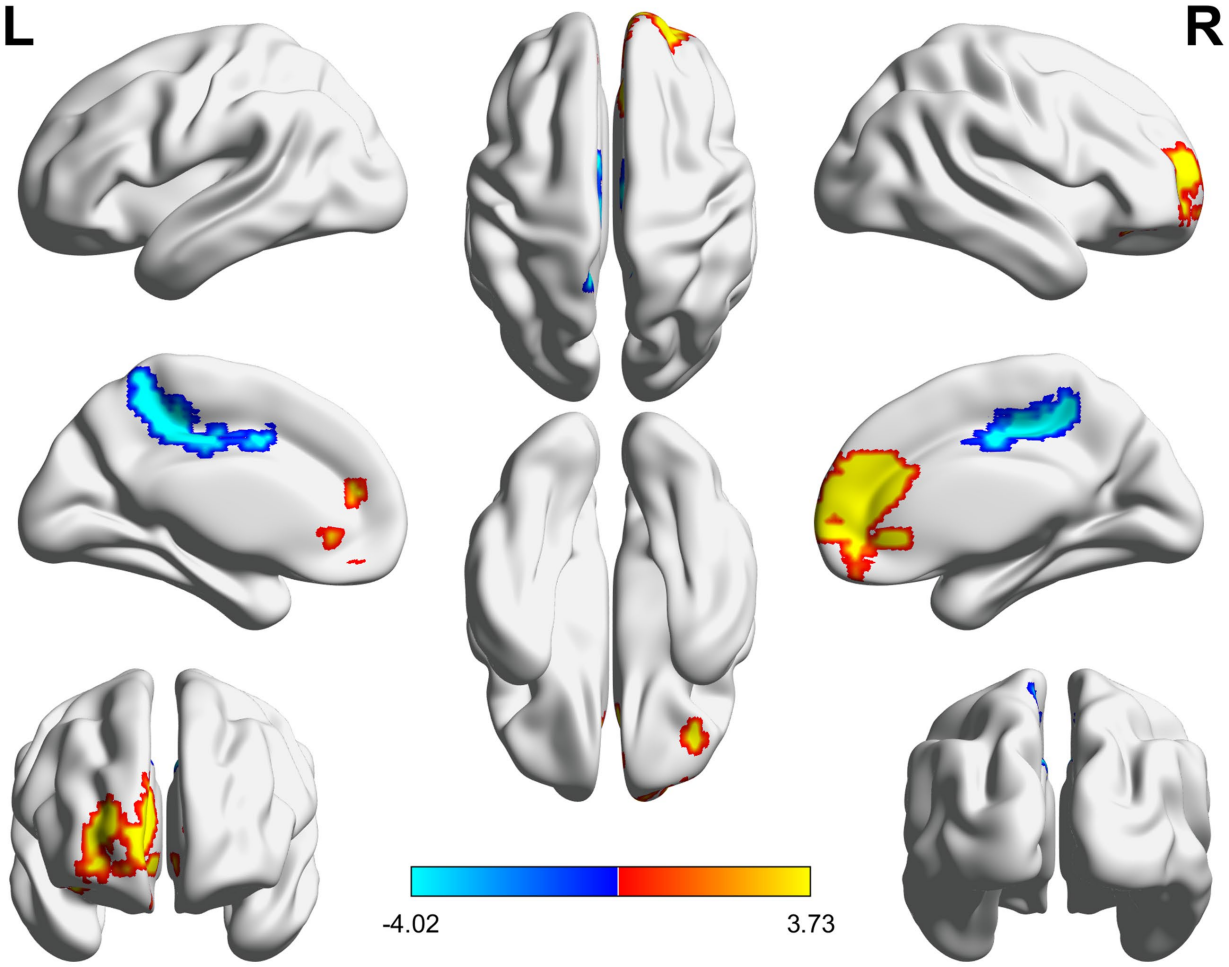
3D brain diagram of the significant changes in the functional connectivity among seed 14 in the true-acupoint group before acupuncture, compared with that HC group. The yellow segments indicate the enhancement in functional connectivity, and the blue segments indicate the reduction in functional connectivity.

**Figure 3:**
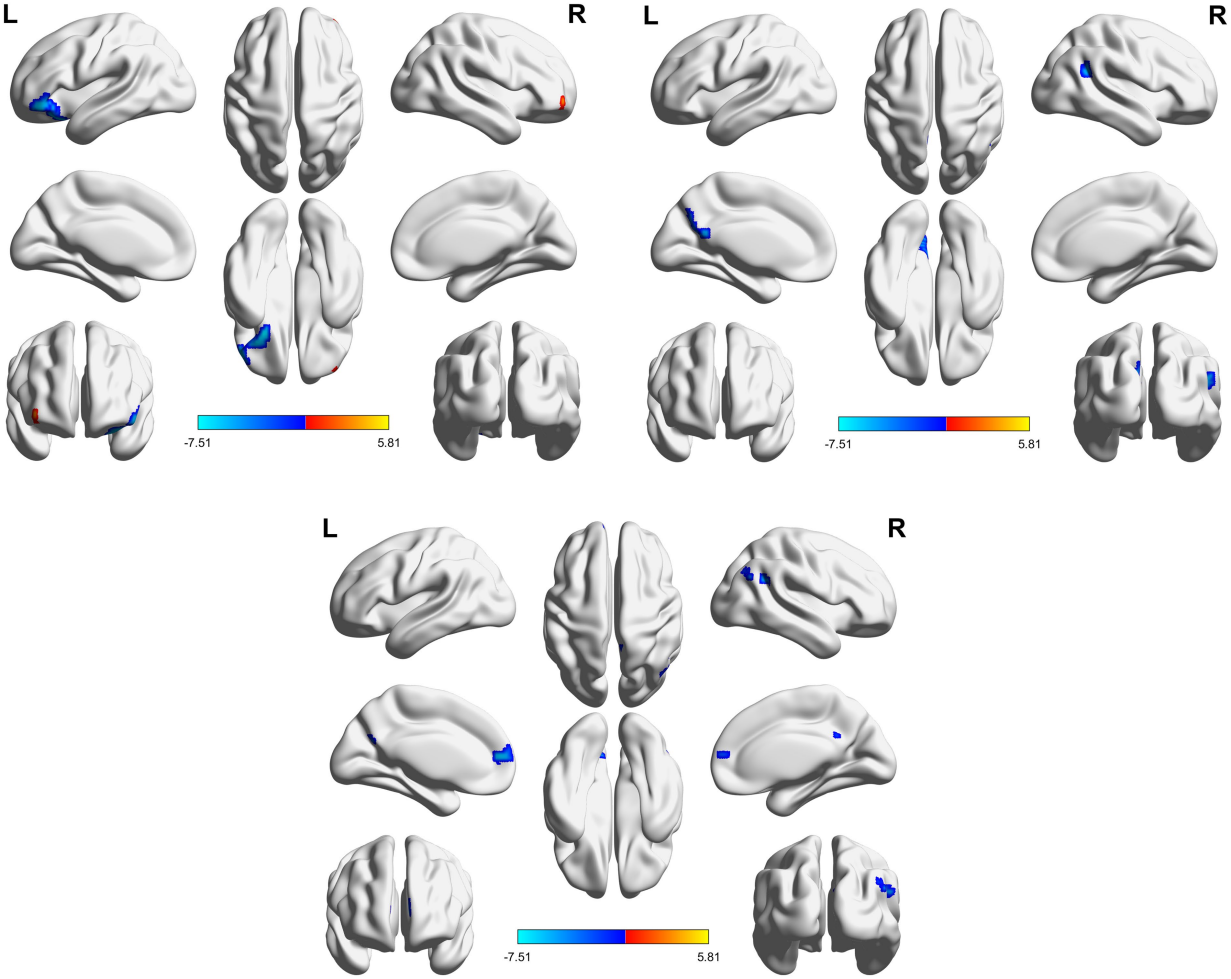
3D brain diagram of the significant changes in the functional connectivity among seed 4 in the true-acupoint group before acupuncture, compared with that HC group. The blue segments indicate the reduction in functional connectivity. The same below. 3D brain diagram of the significant changes in the functional connectivity among seed 29 in the true-acupoint group before acupuncture, compared with that HC group. 3D brain diagram of the significant changes in the functional connectivity among seed 30 in the true-acupoint group before acupuncture, compared with that HC group.

Conduct a between-group statistical analysis of the voxel-wise functional connectivity (FC) with the whole brain. It was found that before the acupuncture intervention, compared with the HC group, only Seed14 in the true-acupoint group of aMCI passed the Family-Wise Error (FEW) correction. When comparing the sham-acupoint group of aMCI with the HC group, Seed4, Seed29, and Seed30 passed the FEW correction. The purpose of the between-group difference comparison is to understand the baseline level differences between aMCI patients in different groups and healthy individuals before acupuncture, providing accurate basic data for the subsequent evaluation of the different effects brought about by acupuncture at true acupoints and sham acupoints. The specific details are shown in Tables 3, 4 and Figures 2, 3.

**Table 3:** Differences in the FC of Seed 14 in the true-acupoint point group before acupuncture, compared with the HC group.

Cluster number	AAL brain regions	*T*	Peak MNI coordinate		
			X	Y	Z
886	Frontal_Sup_Medial_R	8.6353	6	48	12
15	Frontal_Inf_Orb_R	6.4569	36	36	−12
13	Caudate_R	5.772	21	27	15
537	Cingulum_Mid_L	−10.3576	−6	−21	42

The initial voxel-level threshold was set at *p* = 0.000 (FWEc corrected). FC, functional connectivity. A negative *T*-value indicates brain regions with decreased FC, while a positive *T*-value indicates brain regions with increased FC.

**Table 4:** Differences in the FC of the seed points of aMCI in the sham-acupoint point group before acupuncture, compared with the HC group.

Seeds	Cluster number	AAL brain regions	*T*	Peak MNI coordinate		
				X	Y	Z
Seed4 Frontal_Mid_Or_b_R	17	Frontal_Mid_Orb_R	5.8137	42	60	−3
	118	Frontal_Inf_Orb_L	−7.5096	−30	24	−18
Seed29 Temporal_Pole_Mid_L	66	Precuneus_L	−6.3943	−3	−69	30
	17	Angular_R	−5.6882	51	−51	27
Seed30 Temporal_Pole_Mid_R	54	Frontal_Sup_Medial_L	−5.9507	−9	51	9
	21	Precuneus_L	−5.3362	−9	−66	27
	16	Angular_R	−5.3934	54	−51	27

The initial voxel-level threshold was set at *p* = 0.000 (FWEc corrected). FC, functional connectivity. A negative *T*-value indicates brain regions with decreased FC, while a positive *T*-value indicates brain regions with increased FC.

In this experiment, when conducting inter-group statistical analysis before acupuncture in the true acupoint group and the sham acupoint group, it was found that only Seed10, Seed12, and Seed17 passed the Family-Wise Error (FEW) correction. However, due to the small number of voxels, it was impossible to create a graph.

### 3.3 After acupuncture, functional connectivity changes of seed points among the true acupoint group, sham acupoint group, and HC group

**Figure 4:**
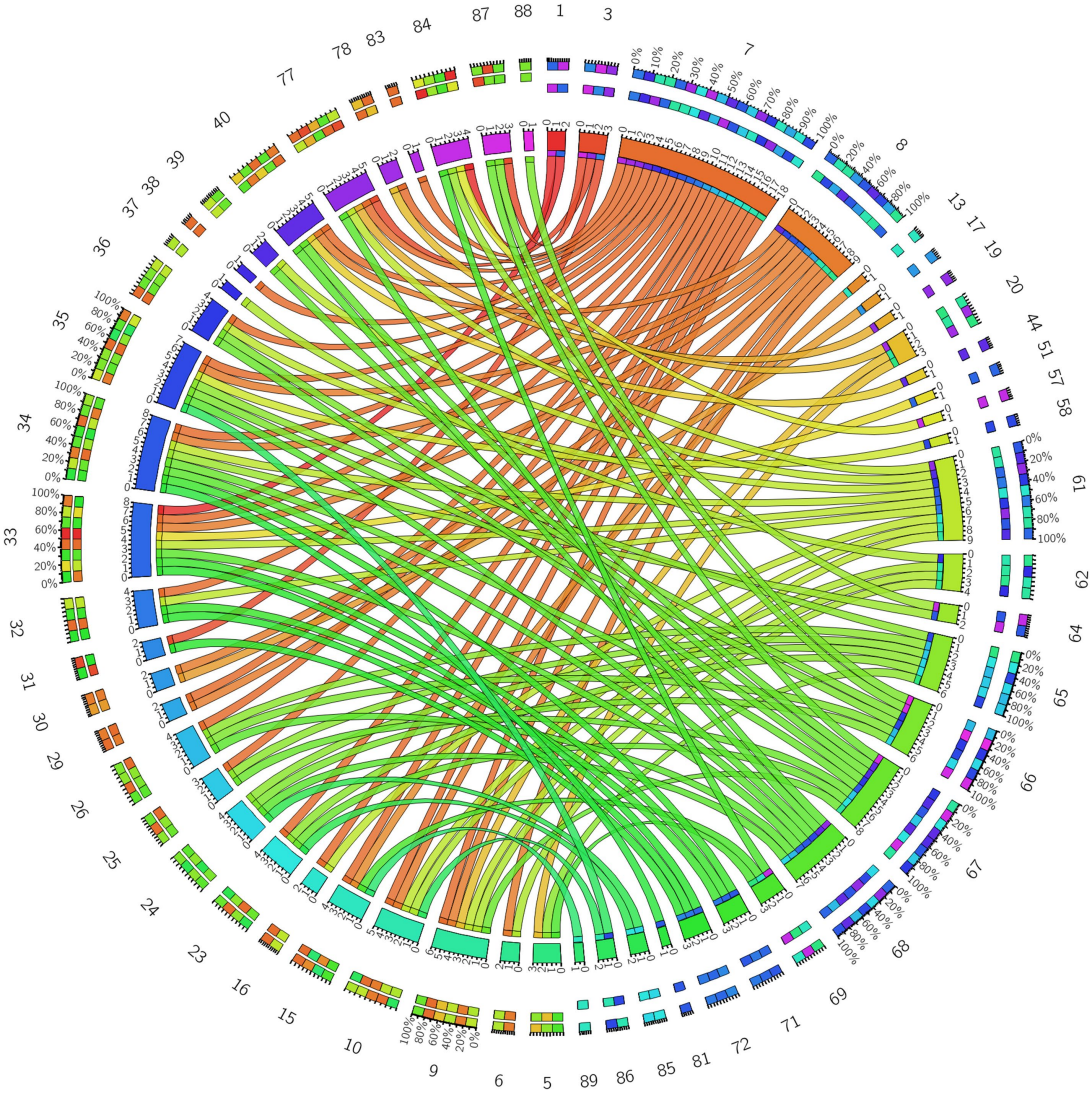
3D brain diagram of the significant changes in the functional connectivity among 30 selected seed points in the ture-acupoint group after acupuncture, compared with that HC group. This Circos plot shows the differences in functional connectivity (FC) between the seed points of the limbic system and other brain regions (corrected by Family-Wise Error, FEW). The full circular area represents the AAL brain regions included in the analysis after FEW correction, and different colors are used to distinguish various brain regions. On the left side of the plot are 30 seed points of the limbic system in the AAL template (arranged in ascending order of serial numbers), and on the right side are the serial numbers of brain regions outside the limbic system. The arcs connect the left and right brain regions, representing the FC changes between the left seed points and the right brain regions. The brain region names corresponding to the serial numbers can be found in the appendix. The same below.

**Figure 5:**
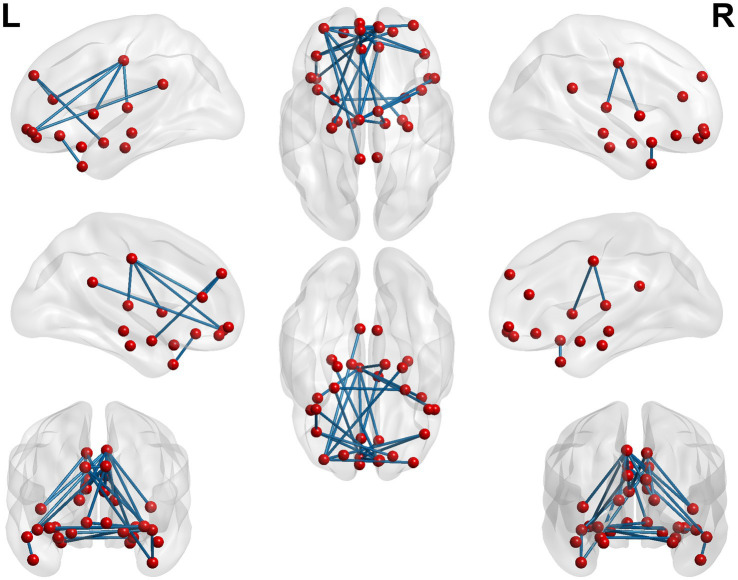
3D brain diagram of the significant changes in the functional connectivity among 30 selected seed points in the ture-acupoint group after acupuncture, compared with that HC group. The blue line segments indicate the reduction in functional connectivity (compared with the HC group).

When comparing the HC group with the true acupoint group after acupuncture, it was found that the changes in FC were mainly characterized by a decrease. Among them, the FC between 18 seed points and the brain region of the left middle frontal gyrus significantly decreased. Followed by that of the right middle frontal gyrus. Next, it was the brain regions below the left parietal bone, excluding the supramarginal gyrus and the angular gyrus. There were also relatively more changes in the middle part of the left cingulate gyrus and the gyri surrounding its lateral side, as well as the bilateral precuneus. The specific details are shown in Table 5 and Figures 4, 5.

**Table 5:** The changes in the FC of the seed points in the true acupoint group after acupuncture, compared with the HC group.

Seeds	Cluster number	AAL brain regions	*T*	Peak MNI coordinate		
				X	Y	Z
**Seed1** Frontal_Sup_Orb_L	73	Angular_L	−6.6116	−42	−66	51
	42	Paracentral_Lobule_L	−6.1548	−9	−45	78
**Seed2** Frontal_Sup_Orb_R	31	Supp_Motor_Area_R	−5.386	0	−12	78
	11	Frontal_Inf_Orb_L	−5.297	−48	42	−9
**Seed3** Frontal_Mid_Orb_L	9	Parietal_Inf_R	−5.283	45	−45	42
	12	Frontal_Mid_R	−5.9068	51	18	48
	39	Cingulum_Mid_L	−5.421	3	−33	48
	222	Parietal_Inf_L	−8.0323	−54	−45	48
	205	Frontal_Mid_L	−7.4305	−27	15	60
	76	Parietal_Inf_R	−6.8438	51	−48	57
	35	Frontal_Mid_R	−5.5685	30	12	63
**Seed4** Frontal_Mid_Orb_R	74	Precuneus_L	−6.6067	−3	−51	72
	62	Supp_Motor_Area_L	−5.8254	−3	−9	78
	35	Frontal_Mid_Orb_L	−5.5889	−39	51	3
	43	Temporal_Mid_R	−5.8473	66	−42	−9
	135	Parietal_Inf_R	−7.296	51	−48	57
	157	Parietal_Inf_L	−7.6673	−54	−45	48
**Seed5** Frontal_Inf_Orb_L	38	Frontal_Mid_L	−6.7342	−27	15	60
	38	Frontal_Sup_R	−5.8685	33	3	63
	27	Temporal_Inf_L	−5.9055	−42	0	−45
	23	Frontal_Inf_Tri_L	−6.2917	−54	30	3
**Seed6** Frontal_Inf_Orb_R	187	Frontal_Sup_Medial_L	−7.0208	0	33	54
	75	Frontal_Mid_L	−7.4568	−42	9	48
	32	Paracentral_Lobule_L	−6.2193	−6	−39	78
**Seed7** Frontal_Sup_Medial_L	39	Frontal_Mid_Orb_L	−6.6287	−42	54	−3
	48	Parietal_Inf_L	−5.637	−54	−57	42
	84	Frontal_Mid_L	−6.5346	−36	24	51
	64	Frontal_Sup_Medial_L	−6.5735	0	36	51
	63	Cerebelum_6_R	−6.1816	21	−81	−30
	42	Temporal_Mid_L	−6.3431	−57	−3	−15
	40	Frontal_Sup_Medial_L	−6.2329	−3	51	18
**Seed8** Frontal_Sup_Medial_R	688	Frontal_Mid_L	−8.1054	−3	42	54
	241	Precuneus_L	−6.5127	−3	−45	33
	238	Angular_L	−6.6357	−48	−66	36
	76	Angular_R	−6.5856	57	−54	33
	64	Cerebelum_Crus1_L	−6.2175	−21	−81	−33
	73	Cerebelum_Crus1_R	−6.3255	21	−81	−30
	66	Temporal_Mid_L	−5.7272	−57	6	−24
	77	Frontal_Sup_Medial_L	−6.6268	0	51	18
**Seed9** Frontal_Med_Orb_L	329	Precuneus_R	−7.1203	12	−48	33
	190	Angular_R	−7.4602	57	−54	33
	164	Angular_L	−5.9877	−39	−69	36
**Seed10** Frontal_Med_Orb_R	439	Frontal_Sup_Medial_L	−7.5512	−3	39	57
	291	Precuneus_L	−6.5575	0	−45	36
	65	Angular_L	−5.6727	−39	−75	45
	139	Frontal_Mid_L	−6.9954	−24	27	48
	15	Hippocampus_R	−6.108	27	−18	−18
	214	Precuneus_R	−6.4228	15	−48	9
**Seed11** Insula_L	52	Angular_R	−6.223	57	−57	30
	41	Angular_L	−6.0484	−39	−72	42
	91	Frontal_Mid_L	−8.2577	−24	27	48
**Seed12** Insula_R	23	Frontal_Sup_Medial_L	−5.45	−6	36	57
	80	Frontal_Mid_L	−7.2258	−33	48	30
	25	Frontal_Mid_R	−5.4577	33	51	24
	57	Cingulum_Mid_L	−6.1704	0	6	45
	249	Cerebelum_8_L	−7.4744	−27	−60	−54
**Seed13** Cingulum_Ant_L	44	Rolandic_Oper_L	−6.3659	−60	9	0
	71	Frontal_Mid_R	−6.1353	63	−30	45
	121	Cingulum_Mid_L	−6.4071	3	3	45
**Seed14** Cingulum_Ant_R	49	Cingulum_Mid_R	−6.21	15	−33	45
	40	Caudate_R	−6.5761	15	9	12
	515	Frontal_Sup_L	−7.2428	−24	48	36
	36	Frontal_Sup_Medial_L	−5.4982	0	42	24
	283	Cingulum_Mid_L	−6.7251	−3	−30	48
**Seed15** Cingulum_Mid_L	55	Caudate_R	−7.6265	15	9	15
	33	Caudate_L	−6.2803	−12	12	15
	596	Frontal_Mid_L	−7.7665	−24	27	51
	78	Frontal_Sup_Medial_L	−5.7107	0	18	36
	169	Cingulum_Mid_L	−5.8693	3	−45	54
	22	Parietal_Inf_L	−5.5217	−54	−57	42
	171	Caudate_L	−6.3863	−15	21	−9
	39	Frontal_Mid_Orb_L	−6.2681	−21	42	−12
	74	Temporal_Pole_Sup_R	−7.088	60	15	−3
	923	Frontal_Mid_L	−9.5793	−27	45	39
	218	Caudate_R	−7.6779	21	24	3
**Seed16** Cingulum_Mid_R	3,740	Precuneus_R	−9.6638	0	−48	60
	46	Thalamus_L	−6.6864	−9	−21	6
	51	Thalamus_R	−5.9282	12	−18	9
	296	Parietal_Inf_L	−6.8698	−60	−39	36
	108	Occipital_Mid_L	−5.8602	−30	−75	39
	264	Frontal_Mid_R	−8.5645	33	45	36
	48	Precentral_L	−6.1302	−48	−9	57
	48	Cerebelum_9_L	−6.1344	−9	−57	−54
	251	Insula_R	−7.5193	30	30	0
	151	Caudate_L	−7.0579	−9	12	9
	82	Precuneus_R	−6.2278	9	−42	6
**Seed17** Cingulum_Post_L	54	Thalamus_R	−6.9686	12	−15	9
	66	Temporal_Sup_R	−6.0582	51	−15	9
	457	Frontal_Mid_L	−7.6731	−27	48	30
	138	Cingulum_Ant_L	−6.4353	0	21	30
	846	Frontal_Mid_R	−9.3675	33	48	33
	57	Parietal_Inf_L	−6.3102	−30	−75	42
	149	SupraMarginal_R	−7.4985	63	−45	45
	972	Precuneus_L	−8.8562	0	−48	57
	17	Cerebelum_9_R	−5.6315	9	−54	−42
	37	Cerebelum_Crus1_R	−5.6933	39	−69	−39
**Seed18** Cingulum_Post_R	25	Cerebelum_Crus2_L	−6.0425	−24	−81	−33
	16	Temporal_Mid_R	−5.4046	63	0	−15
	69	Frontal_Med_Orb_L	−6.1537	−3	63	−3
**Seed19** Hippocampus_L	861	Frontal_Mid_L	−8.1202	−24	30	51
**Seed20** Hippocampus_R	570	Precuneus_R	−8.9735	0	−63	45
**Seed21**	362	Angular_L	−7.2443	−39	−66	48
ParaHippocampal_L	116	Angular_R	−6.898	57	−54	27
**Seed22** ParaHippocampal_R	15	Frontal_Mid_R	−5.9943	48	36	33
	45	Postcentral_R	−5.9674	42	−33	63
	152	Parietal_Inf_L	−6.3254	−30	−75	45
	431	Precuneus_L	−8.3755	0	−30	42
	38	Frontal_Mid_L	−5.8034	−24	27	54
	49	Angular_R	−5.9097	51	−57	48
**Seed23** Amygdala_L	25	Parietal_Inf_L	−5.5346	−30	−78	48
**Seed25** Thalamus_L	4	Frontal_Mid_L	−5.1349	−21	24	51
	20	Parietal_Inf_L	−5.4593	−33	−72	42
	18	Precuneus_L	−5.3086	0	−63	57
	19	Frontal_Mid_Orb_L	−6.0452	−45	48	−9
**Seed26** Thalamus_R	46	Calcarine_R	−6.0634	21	−54	15
	49	Precuneus_R	−6.7693	3	−72	36
	24	Frontal_Mid_L	−5.4758	−24	30	45
**Seed27** Temporal_Pole_Sup_L	23	Precuneus_L	−6.0744	0	−63	57
	18	Frontal_Mid_R	−5.7213	33	6	66
**Seed28** Temporal_Pole_Sup_R	13	Amygdala_R	−5.7423	21	6	−18
	13	Frontal_Sup_Medial_L	−5.4929	−6	36	54
	33	Frontal_Mid_L	−5.2825	−30	60	15
	438	Precuneus_R	−6.6041	3	−36	48
	49	Parietal_Inf_L	−5.6828	−45	−48	51
	46	Frontal_Sup_L	−6.2538	−18	6	69
**Seed29** Temporal_Pole_Mid_L	84	Supp_Motor_Area_L	−6.8757	0	−9	78
	89	Cingulum_Mid_L	−6.8087	3	−36	48
	26	Frontal_Mid_L	−5.6336	−27	36	45
	79	Supp_Motor_Area_R	−7.2988	6	−6	78
**Seed30** Temporal_Pole_Mid_R	19	Frontal_Mid_L	−5.3686	−30	45	30
	14	Temporal_Pole_Sup_R	6.6013	51	18	−21
	14	SupraMarginal_L	−6.5187	−54	−21	18
	24	Cingulum_Mid_L	−5.5805	−6	−12	42
	21	Postcentral_L	−6.6257	−54	−12	48
	18	Precentral_L	−5.9445	−45	−12	60
	22	Paracentral_Lobule_L	−6.9365	−18	−27	78
	21	Frontal_Inf_Orb_L	−5.8988	−51	33	−3
	110	Frontal_Sup_L	−6.0941	0	60	18
	24	Angular_R	−5.7209	51	−48	27
	200	Precuneus_L	−6.138	0	−69	33
	36	Frontal_Sup_Medial_R	−5.6916	3	36	51
	41	Angular_R	−6.0713	60	−54	27

The initial voxel-level threshold was set at *p* = 0.000 (FWEc corrected). FC, functional connectivity. A negative T-value indicates brain regions with decreased FC, while a positive *T*-value indicates brain regions with increased FC.

**Figure 6:**
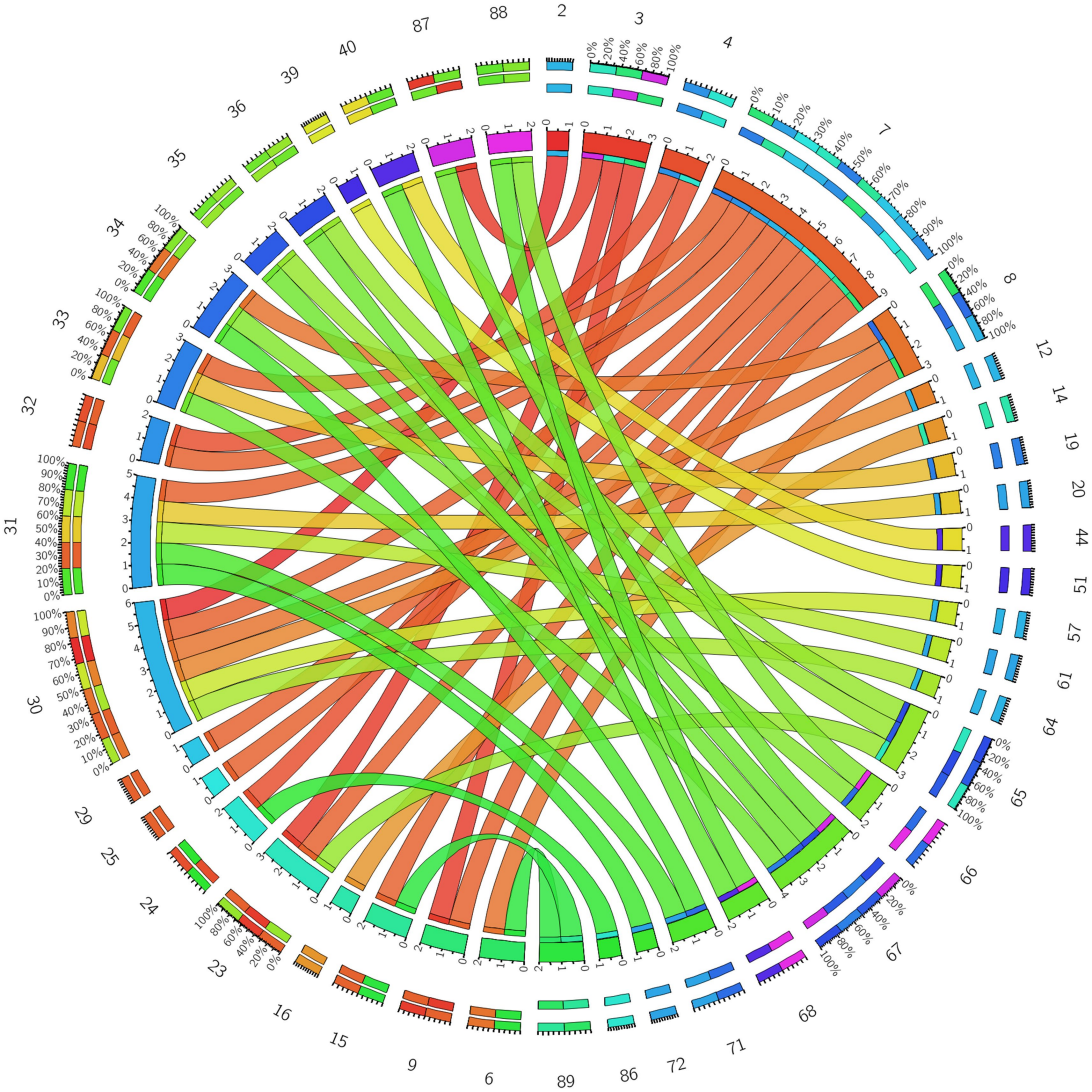
Circos diagram of the significant changes in the functional connectivity between 30 select seed points and the brain regions of the AAL template in the whole brain in the sham-acupoint group after acupuncture, compared with that HC group.

**Figure 7:**
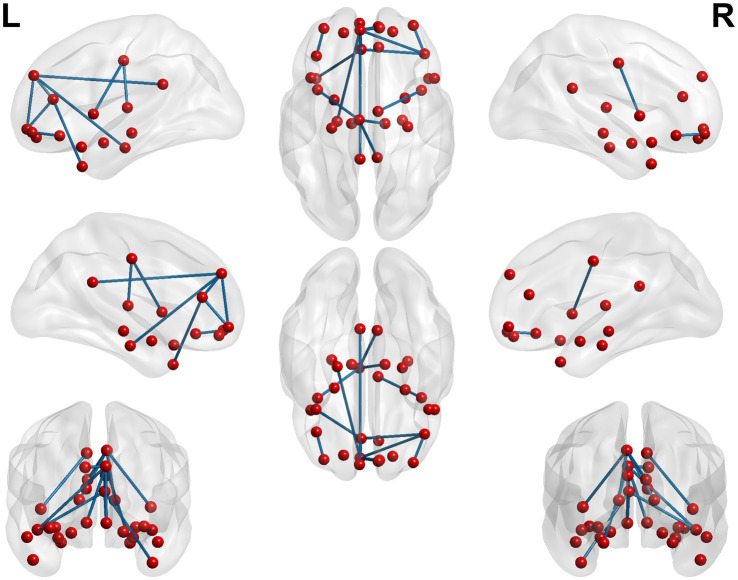
3D brain diagram of the significant changes in the functional connectivity among 30 selected seed points in the sham-acupoint group after acupuncture, compared with that HC group. The blue line segments indicate the reduction in functional connectivity.

However, when comparing the HC group with the sham acupoint group after acupuncture, it was found that the changes in FC were mainly characterized by a decrease. Among them, the number of seed points with a decreased FC to the left middle frontal gyrus was the largest, but it was less than that in the true acupoint group. Secondly, the functional connectivity of multiple seed points with the left medial superior frontal gyrus (Seed7) significantly decreased. In addition, there were also relatively more changes in other regions such as the left precuneus, the middle part of the left cingulate gyrus and the gyri surrounding its lateral side. The specific details are shown in Table 6 and Figures 6, 7.

**Table 6:** The changes in the FC of the seed points in the sham-acupoint group after acupuncture, compared with the HC group.

Seeds	Cluster number	AAL brain regions	*T*	Peak MNI coordinate		
				X	Y	Z
Seed2 Frontal_Sup_Orb_R	9	Temporal_Inf_L	−6.1372	−42	9	−42
	34	Frontal_Mid_R	−6.1248	51	18	42
Seed3 Frontal_Mid_Orb_L	24	Frontal_Inf_Orb_L	−6.0099	−42	48	−12
	26	Frontal_Sup_L	−6.0153	−18	−12	60
	20	Frontal_Mid_L	−6.1679	−24	15	60
Seed4 Frontal_Mid_Orb_R	51	Frontal_Inf_Orb_R	−7.6907	45	48	−12
Seed5 Frontal_Inf_Orb_L	22	Temporal_Inf_L	−5.9155	−42	6	−45
	20	Frontal_Mid_L	−7.031	−42	12	48
Seed6 Frontal_Inf_Orb_R	50	Cingulum_Ant_L	−5.9741	−6	48	12
	28	Frontal_Inf_Tri_R	−6.4913	51	24	27
	44	Frontal_Sup_Medial_L	−6.7816	0	36	51
Seed7 Frontal_Sup_Medial_L	35	Cerebelum_Crus1_R	−6.2255	21	−81	−30
	65	Angular_L	−5.9661	−60	−57	27
	104	Frontal_Mid_L	−7.9604	−42	12	51
	238	Frontal_Sup_L	−7.2434	−9	36	57
Seed8 Frontal_Sup_Medial_R	35	Temporal_Mid_R	−5.7782	60	−15	−12
	184	Frontal_Sup_Medial_L	−7.43	−9	36	57
	46	Frontal_Sup_R	−5.9285	18	42	54
Seed9 Frontal_Med_Orb_L	9	Cingulum_Ant_L	−6.2175	−3	45	3
	19	Frontal_Sup_Medial_L	−6.3255	−9	69	21
	14	Frontal_Mid_L	−5.1849	−27	27	48
Seed10 Frontal_Med_Orb_R	7	Frontal_Sup_Medial_L	−6.6268	0	39	51
	17	Cerebelum_3_R	−6.1678	9	−30	−27
Seed11 Insula_L	91	Cingulum_Mid_L	−7.4524	−6	15	39
	110	Frontal_Mid_L	−8.3855	−33	48	30
Seed12 Insula_R	21	Cerebelum_8_L	−6.0169	−24	−66	−48
	20	Frontal_Inf_Oper_R	−5.6804	51	12	21
	63	Frontal_Mid_R	−6.9691	33	48	30
	113	SupraMarginal_R	−7.7541	60	−30	30
	20	Frontal_Mid_L	−6.1446	−33	42	30
	32	Cingulum_Mid_R	−6.2565	18	−36	42
	19	Postcentral_L	−5.4257	−45	−9	51
	39	Precentral_R	−5.9565	51	9	51
Seed13 Cingulum_Ant_L	103	Frontal_Med_Orb_L	−6.6142	6	51	−6
	31	Caudate_R	−6.1072	15	18	6
	16	Caudate_L	−5.7443	−15	21	6
	192	Frontal_Mid_L	−7.6662	−24	48	36
	23	Parietal_Inf_L	−5.9266	−57	−51	36
	51	Frontal_Sup_Medial_R	−6.4204	12	48	45
	28	Supp_Motor_Area_R	−6.1149	12	15	66
Seed14 Cingulum_Ant_R	109	Frontal_Mid_L	−6.6666	−24	48	33
	64	Frontal_Sup_R	−6.2309	18	48	42
Seed15 Cingulum_Mid_L	80	Thalamus_R	−6.1399	21	−18	12
	63	Frontal_Mid_L	−6.3574	−45	48	9
	389	Precuneus_L	−6.9489	−15	−60	27
	182	Supp_Motor_Area_L	−6.328	−3	24	30
Seed16 Cingulum_Mid_R	24	Caudate_L	−5.8919	−24	21	6
	28	Angular_R	−7.3033	42	−72	36
	20	Frontal_Mid_R	−6.0027	39	3	60
Seed17 Cingulum_Post_L	26	Frontal_Sup_Medial_L	−6.0139	−6	69	18
	156	Precuneus_L	−6.677	−3	−66	36
	21	Angular_L	−5.7253	−42	−69	42
Seed18 Cingulum_Post_R	78	Precuneus_L	−6.2224	0	−66	36
	21	Angular_L	−5.503	−36	−75	39
	34	Cingulum_Mid_L	−6.0701	0	−27	42
Seed21 ParaHippocampal_L	10	Frontal_Sup_Medial_L	−5.6499	−6	66	15
	18	Occipital_Mid_L	−6.369	−39	−72	36
Seed22 ParaHippocampal_R	32	Calcarine_R	−6.6198	21	−54	18
	130	Precuneus_R	−7.6081	6	−72	36
Seed25 Thalamus_L	17	Cingulum_Mid_L	−5.7407	−9	−27	39
Seed26 Thalamus_R	42	Cingulum_Mid_L	−6.0394	0	−21	39
Seed29 Temporal_Pole_Mid_L	93	Cingulum_Ant_L	−6.3574	−9	48	6
	7	Frontal_Sup_L	−5.5192	−18	57	24
	33	Precuneus_L	−6.5173	0	−69	33
Seed30 Temporal_Pole_Mid_R	36	Angular_R	−6.1815	48	−72	36
	22	Precuneus_R	−5.4932	0	−48	30

The initial voxel-level threshold was set at *p* = 0.000 (FWEc corrected). FC, functional connectivity. A negative *T*-value indicates brain regions with decreased FC, while a positive *T*-value indicates brain regions with increased FC.

In this experiment, when conducting statistical analysis on the inter - group FC after acupuncture in the true and the sham acupoint group, we found that not only Seed10 and Seed12 passed the Family - Wise Error (FEW) correction, but also Seed2, Seed11, Seed14, Seed19, Seed24, and Seed25 passed the FEW correction, showing significant differences. However, due to the extremely small number of voxels, it was impossible to plot relevant graphs.

### 3.4 The changes in the FC of the seed points in the true acupoint group and the sham acupoint group before and after acupuncture

**Figure 8:**
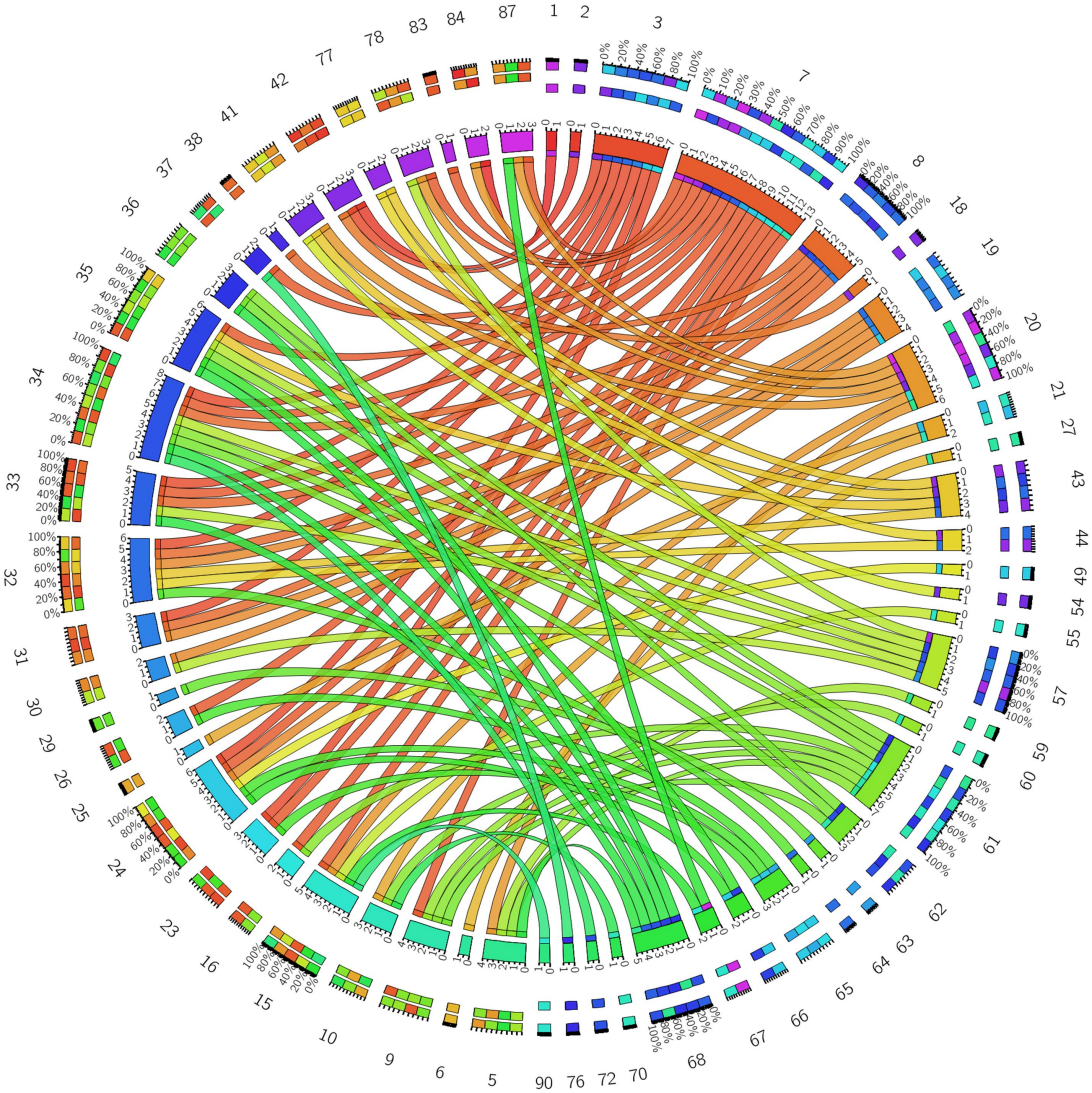
Circos diagram of the significant changes in the functional connectivity between 30 selected seed points and the brain regions of the AAL template in the whole brain in the true-acupoint group after acupuncture, compared with that before acupuncture.

**Figure 9:**
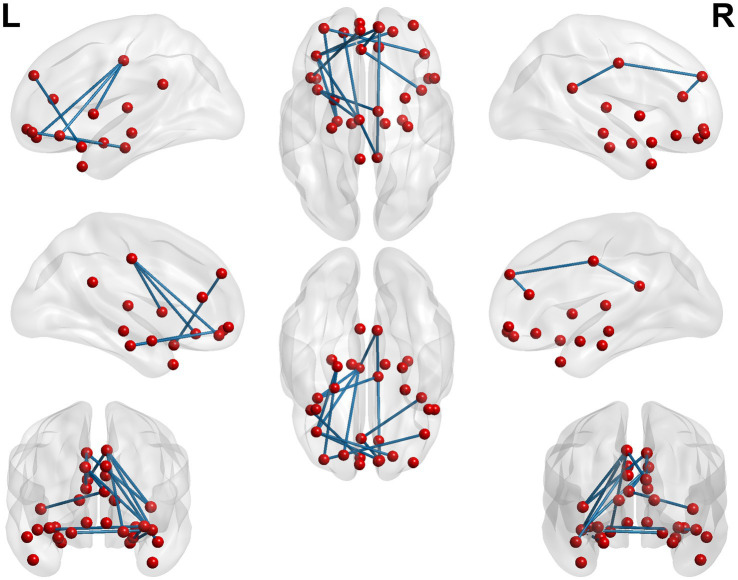
3D brain diagram of the significant changes in the functional connectivity among 30 selected seed points in the true-acupoint group after acupuncture, compared with that before acupuncture. The blue line segments indicate the reduction in functional connectivity.

When conducting the within-group comparison before and after acupuncture in the true acupoint group, it was found that the changes in FC were mainly characterized by a decrease. Among them, the seed points with the most significant decrease in FC were those connected to the left middle frontal gyrus. Secondly, it was the brain regions below the left parietal bone, excluding the supramarginal gyrus and the angular gyrus. Thirdly, it was the right middle frontal gyrus and the right precuneus. The specific details are shown in Table 7 and Figures 8, 9.

**Table 7:** The changes in the FC of the seed points within the true acupoint group before and after acupuncture.

Seeds	Cluster number	AAL brain regions	*T*	Peak MNI coordinate		
				X	Y	Z
Seed1 Frontal_Sup_Orb_L	16	Cerebelum_8_L	−6.7503	−27	−54	−54
	18	Cingulum_Mid_L	−6.5021	−3	−3	45
	19	Parietal_Inf_L	−7.2924	−51	−54	48
	52	Parietal_Sup_L	−7.0631	−33	−69	57
	23	Precuneus_R	−7.7096	12	−81	51
	34	Supp_Motor_Area_R	−6.8272	−3	−39	78
Seed2 Frontal_Sup_Orb_R	12	Cerebelum_8_L	−6.8941	−27	−48	−54
	49	Frontal_Inf_Orb_L	−6.6887	−24	42	−15
	11	Rectus_R	−6.5711	9	30	−12
Seed3 Frontal_Mid_Orb_L	167	Parietal_Inf_L	−7.6702	−51	−54	45
	65	Parietal_Sup_R	−6.915	45	−57	57
	24	Parietal_Inf_R	−6.6128	60	−51	45
	78	Frontal_Mid_L	−7.0452	−33	24	48
Seed4 Frontal_Mid_Orb_R	72	Olfactory_L	−6.1337	9	27	−12
	180	Parietal_Inf_L	−6.4913	−36	−69	57
	30	Paracentral_Lobule_R	−6.7816	6	−36	78
Seed5 Frontal_Inf_Orb_L	23	Fusiform_L	−7.0726	−30	3	−45
	14	Temporal_Inf_R	−6.0956	48	6	−42
	98	Frontal_Mid_L	−8.8373	−42	15	45
	47	Frontal_Sup_Medial_R	−7.4269	6	45	48
	13	Precuneus_L	−7.109	−3	−45	78
	17	Supp_Motor_Area_R	−5.9222	0	−9	78
Seed6 Frontal_Inf_Orb_R	34	Frontal_Mid_Orb_L	−6.5468	−42	51	−6
	119	Parietal_Inf_L	−7.0364	−51	−63	39
	147	Frontal_Mid_L	−7.4082	−45	15	48
Seed7 Frontal_Sup_Medial_L	542	Frontal_Sup_L	−9.2162	0	12	69
	34	Angular_L	−6.108	−48	−63	36
	118	Frontal_Mid_L	−7.1161	−48	15	45
Seed8 Frontal_Sup_Medial_R	30	Occipital_Sup_L	−7.0831	−9	−102	9
	44	Frontal_Sup_L	−6.7473	−24	69	12
	51	Angular_L	−6.1802	−48	−63	36
	73	Angular_R	−6.6981	57	−54	36
	279	Supp_Motor_Area_L	−7.5898	0	12	69
	30	Cingulum_Mid_R	−6.9188	3	−18	42
	20	Frontal_Mid_L	−5.7034	−42	18	45
Seed10 Frontal_Med_Orb_R	18	Olfactory_L	−6.0169	−3	12	−15
	9	Cerebelum_4_5_L	−7.3435	−6	−4	0
	43	Angular_L	−6.9765	−42	−72	48
	53	Frontal_Mid_L	−6.3496	−24	24	51
Seed11 Insula_L	63	SupraMarginal_L	−7.5116	−60	−24	24
Seed12 Insula_R	17	Cingulum_Ant_L	−6.3262	−3	36	18
	15	Postcentral_L	−5.7731	−48	−12	42
	16	Supp_Motor_Area_L	−6.5654	0	3	75
Seed13 Cingulum_Ant_L	19	Frontal_Sup_Medial_R	−5.7059	6	69	6
	52	Frontal_Sup_L	−6.675	−27	60	12
	80	Frontal_Mid_R	−6.6422	27	60	18
	206	Supp_Motor_Area_L	−7.9108	−12	18	66
Seed14 Cingulum_Ant_R	20	Frontal_Sup_Medial_R	−5.9788	0	66	−6
	92	Frontal_Sup_L	−6.7294	−27	60	12
	23	Caudate_L	−6.7492	−12	15	15
	29	Caudate_R	−9.2777	18	9	15
	22	SupraMarginal_R	−6.0941	60	−48	30
	40	Frontal_Mid_R	−6.4158	30	42	42
	394	Supp_Motor_Area_L	−8.385	0	12	72
Seed15 Cingulum_Mid_L	27	Insula_L	−6.6408	−30	18	12
	878	Frontal_Mid_L	−10.3278	−27	45	39
	35	Cingulum_Post_R	−6.4243	6	−39	24
	119	Frontal_Inf_Oper_R	−6.6462	45	18	27
	55	Frontal_Mid_R	−5.851	39	48	30
	1,215	Precuneus_R	−9.3451	3	−48	57
	279	Postcentral_L	−7.0418	−24	−27	66
	26	Frontal_Sup_L	−6.057	−15	−12	72
Seed16 Cingulum_Mid_R	27	Insula_L	−6.7415	−27	27	6
	31	Frontal_Sup_L	−6.3365	−30	66	6
	1,133	Frontal_Mid_L	−10.1931	30	3	66
	28	Caudate_R	−6.3024	18	−9	18
	118	Frontal_Mid_R	−7.6122	30	57	30
	23	Frontal_Sup_Medial_R	−6.2019	3	42	36
	204	Parietal_Inf_L	−8.3223	−39	−60	54
	113	Parietal_Inf_R	−7.0935	48	−57	54
	268	Precuneus_R	−7.7529	12	−66	60
	16	Postcentral_L	−7.1105	−18	−42	72
Seed17 Cingulum_Post_L	24	Calcarine_L	−5.8577	−9	−99	0
	25	Angular_R	−6.3691	57	−57	33
	210	Precuneus_R	−6.8289	3	−63	33
	54	Frontal_Mid_L	−6.3492	−45	18	45
	112	Parietal_Inf_L	−7.1474	−39	−60	54
	98	Postcentral_L	−7.0026	−45	−33	57
Seed18 Cingulum_Post_R	32		−6.0879	3	−63	45
	47	Cingulum_Mid_R	−6.448	3	−27	42
	43	Parietal_Inf_L	−6.4097	−39	−63	54
	38	Parietal_Inf_R	−6.8636	4	−60	54
Seed19 Hippocampus_L	9	Pallidum_R	5.6666	27	−3	−9
	15	Frontal_Mid_L	−6.4582	−30	15	60
Seed20 Hippocampus_R	25	Frontal_Mid_R	−6.4952	33	6	66
Seed22 Hippocampus_R	15	Frontal_Mid_Orb_L	−7.2619	−45	48	−9
	21	Frontal_Inf_Orb_L	−6.1828	−45	24	−9
Seed23 Amygdala_L	19	Supp_Motor_Area_R	−5.8494	6	−9	75
	10	Occipital_Inf_R	−5.3801	42	−78	−3
	10	Calcarine_L	−5.4556	−6	−42	3
Seed24 Amygdala_R	22	Rolandic_Oper_R	−6.3397	45	−6	18
	17	Precentral_R	−6.531	60	0	42
	74	Frontal_Sup_L	−6.7186	−12	39	48
Seed25 Thalamus_L	94	Calcarine_L	−8.5358	−12	−99	−6
	23	Calcarine_R	−6.3328	18	−99	3
Seed26 Thalamus_R	99	Frontal_Mid_L	−7.1068	−45	15	48
	160	Supp_Motor_Area_R	−8.8552	6	−6	78
	21	Postcentral_L	−6.4977	−21	−30	75
Seed27 Temporal_Pole_Sup_L	36	Frontal_Mid_L	−6.4882	−42	15	45
	18	Frontal_Sup_Medial_R	−6.0649	6	45	51
Seed28 Temporal_Pole_Sup_R	81	Precentral_L	−8.2383	−36	−3	63
	17	Supp_Motor_Area_R	−5.944	6	−6	75
Seed29 Temporal_Pole_Mid_L	11	Frontal_Mid_L	−6.2832	−39	12	48
	45	Supp_Motor_Area_R	−7.4851	0	−9	78
	10	Precuneus_L	−6.3547	−6	−42	78

The initial voxel-level threshold was set at *p* = 0.000 (FWEc corrected). FC, functional connectivity. A negative *T*-value indicates brain regions with decreased FC, while a positive *T*-value indicates brain regions with increased FC.

**Figure 10:**
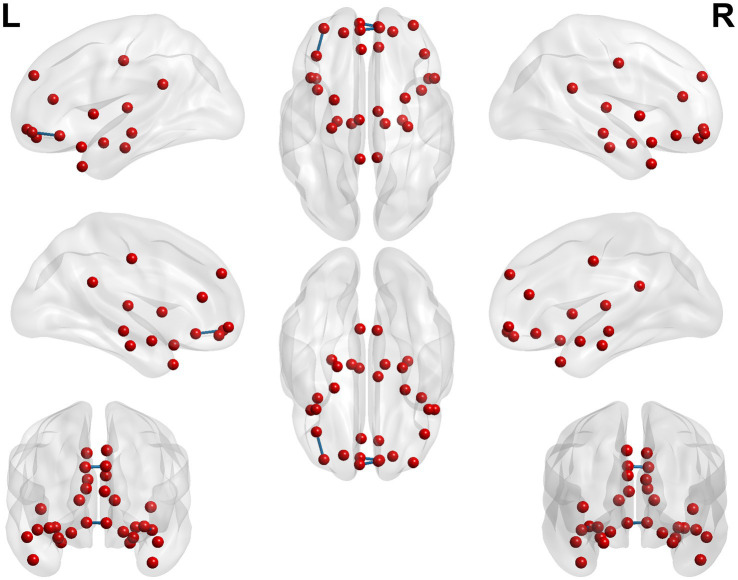
3D brain diagram of the significant changes in the functional connectivity among 30 selected seed points in the sham-acupoint group after acupuncture, compared with that before acupuncture. The blue line segments indicate the reduction in functional connectivity (compared with the HC group).

When conducting the within-group comparison before and after acupuncture in the sham acupoint group, it was found that the functional connectivity of multiple seed points did not pass the Family-Wise Error (FEW) correction. Only a few seed points had brain regions with significant differences, including Seed5, Seed8, Seed10, and Seed15. The specific details are shown in Table 8 and Figure 10.

**Table 8:** The changes in the FC of the seed points within the sham acupoint group before and after acupuncture.

Seeds	Cluster number	AAL brain regions	*T*	Peak MNI coordinate		
				X	Y	Z
Seed5 rontal_Inf_Orb_L	6	Frontal_Mid_Orb_L	−5.2059	−33	27	−3
Seed8 Frontal_Sup_Medial_R	5	Frontal_Sup_Medial_	−4.1024	0	60	36
Seed10 Frontal_Med_Orb_R	5	Frontal_Med_Orb_L	−5.0104	−3	45	−12
Seed15 Cingulum_Mid_L	7	Precentral_L	−8.0401	−42	−6	48

The initial voxel-level threshold was set at *p* = 0.000 (FWEc corrected). FC, functional connectivity. A negative *T*-value indicates brain regions with decreased FC, while a positive *T*-value indicates brain regions with increased FC.

### 3.5 Statistical analysis and correlation analysis of the functional connectivity values of the seed points in the HC group, the true-acupoint group, and the sham-acupoint group

In this study, after performing inter - group and intra - group t - tests using the SPM12 software, the FC values of significant brain regions were extracted based on voxel size. Subsequently, the IBM SPSS V25.0 software was employed to conduct one - way analysis of variance (ANOVA) on the FC values of the HC group, the pre - acupuncture and the post - acupuncture in the true - acupoint group, as well as the HC, the pre - acupuncture and the post - acupuncture in the sham - acupoint group, respectively. Among them, when comparing the true-acupoint group, the FC values of Seed3, Seed4, Seed7, Seed8, Seed13, Seed14, Seed15, Seed16, Seed17, Seed18, Seed25, Seed29 with the bilateral middle frontal gyrus, the left medial superior frontal gyrus, the middle part of the left cingulate gyrus and the gyri surrounding its side, the gyri below the bilateral parietal bones except the supramarginal gyrus and the angular gyrus, as well as the bilateral precuneus were extracted. When comparing the sham-acupoint group, the FC values of Seed7, Seed8, Seed13, Seed15, Seed17 with the left middle frontal gyrus, the left medial superior frontal gyrus, and the left precuneus were extracted.

**Figure 11:**
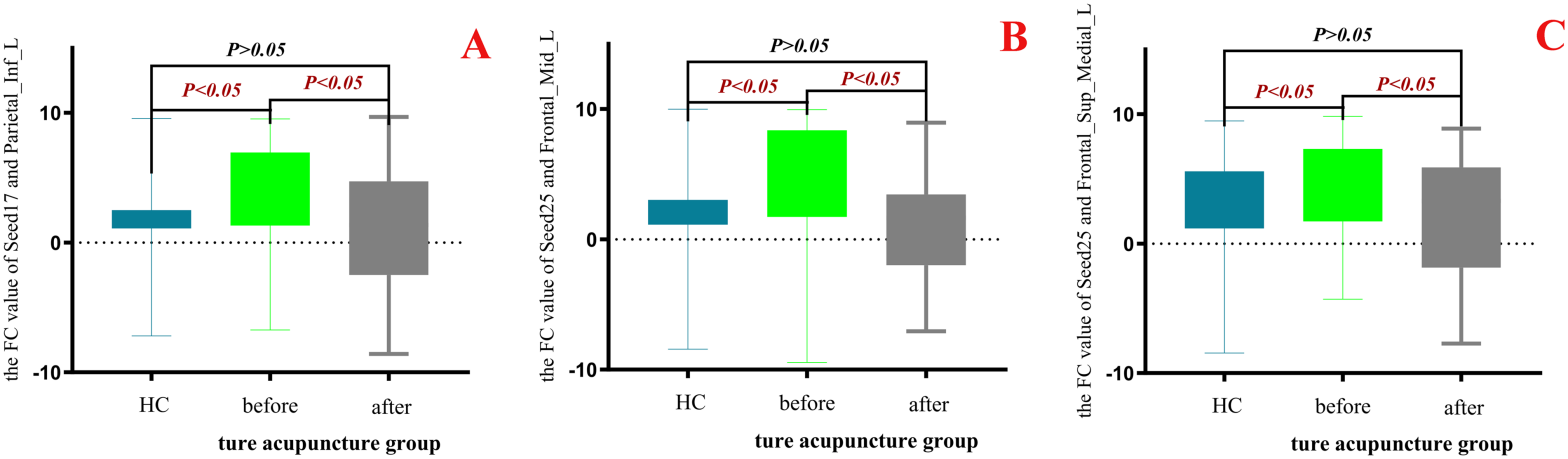
Box - plot of the results of one - way ANOVA for FC values in the HC group, true - acupoint group before and after acupuncture. *p* < 0.05 indicates statistical significance, which is marked in red font. Blue represents the healthy control group (HC group), green represents the true-acupoint group before acupuncture, and gray represents the true-acupoint group after acupuncture. **(A)** shows the comparisons of the FC values between Seed17 and Parietal_Inf_L; **(B)** shows the comparisons of the FC values between Seed25 and Frontal_Mid_L; **(C)** shows the comparisons of the FC values between Seed25 and Frontal_Sup_Medial_L.

The results showed that no effective changes in the FC values between brain regions were detected in the data analysis with the sham - acupoint group. However, in the data analysis with the true - acupoint group, significant differences were found in the FC values of Seed17 - Parietal_Inf_L, Seed25 - Frontal_Mid_L, and Seed25 - Frontal_Sup_Medial_L. Finally, the Prism10.1.2 software was used to visualize the data results, to display the differences in FC values among different groups and analyze the effect of acupuncture on the functional connectivity of relevant brain regions, as shown in Figure 11.

In this experiment, a correlation analysis was carried out between the FC values before acupuncture in the true - acupoint group and the scores of neuropsychological scales. The results showed that multiple brain regions had a significant correlation with the scores of the MMSE and the MoCA. Specifically, the FC values of Seed16 - Seed15 and Seed13 - Parietal_Inf_L were significantly negatively correlated with both the MMSE and MoCA scores. In addition, it was found that the MoCA score was correlated with the FC values of more seed points, mainly negative correlation, indicating that it is more representative in characterizing the abnormal functional connectivity between brain regions in patients with aMCI. See Table 9 for details.

**Table 9:** Table of correlation analysis between FC values of seed points and other brain regions and neuropsychological scale scores in the true - acupoint group before acupuncture.

Functional connectivity (FC)	Neuropsychological scale score	Pearson correlation	
		*r*	*P*
Seed15- Parietal_Inf_R	MMSE	0.339	0.046
Seed16- Parietal_Inf_R	MMSE	0.366	0.031
Seed29- Frontal_Mid_R	MMSE	0.342	0.044
Seed13- Parietal_Inf_L	MMSE	−0.349	0.040
Seed16-Seed15	MMSE	−0.420	0.012
Seed15- Parietal_Inf_L	MoCA	0.362	0.033
Seed3- Frontal_Mid_R	MoCA	−0.355	0.037
Seed3- Parietal_Inf_R	MoCA	−0.352	0.038
Seed3- Precuneus_L	MoCA	−0.420	0.012
Seed3- Precuneus_R	MoCA	−0.345	0.042
Seed4- Parietal_Inf_L	MoCA	−0.462*	0.005*
Seed4- Parietal_Inf_R	MoCA	−0.409	0.015
Seed4- Precuneus_L	MoCA	−0.340	0.046
Seed7- Frontal_Mid_L	MoCA	−0.445*	0.007*
Seed7- Frontal_Mid_R	MoCA	−0.637*	0.000*
Seed7- Parietal_Inf_L	MoCA	−0.426	0.011
Seed7- Parietal_Inf_R	MoCA	−0.407	0.015
Seed8- Frontal_Mid_L	MoCA	−0.525*	0.001*
Seed8- Frontal_Mid_R	MoCA	−0.568*	0.000*
Seed8- Parietal_Inf_L	MoCA	−0.504*	0.002*
Seed8- Parietal_Inf_R	MoCA	−0.487	0.003
Seed8- Precuneus_L	MoCA	−0.376	0.026
Seed8- Precuneus_R	MoCA	−0.351	0.038
Seed13- Frontal_Mid_L	MoCA	−0.443*	0.008*
Seed13- Frontal_Mid_R	MoCA	−0.521*	0.001*
Seed13- Frontal_Sup_Medial_L	MoCA	−0.367	0.030
Seed13- Parietal_Inf_L	MoCA	−0.505*	0.002*
Seed13- Parietal_Inf_R	MoCA	−0.447*	0.007*
Seed13- Precuneus_L	MoCA	−0.431*	0.010*
Seed13- Precuneus_R	MoCA	−0.366	0.031
Seed14- Frontal_Mid_L	MoCA	−0.477*	0.004*
Seed14- Frontal_Mid_R	MoCA	−0.453*	0.006*
Seed14- Parietal_Inf_R	MoCA	−0.360	0.034
Seed15- Precuneus_R	MoCA	−0.520*	0.001*
Seed16-Seed15	MoCA	−0.347	0.041

*, It indicates that the correlation is significant at the 0.01 level (two-tailed); The remaining data indicate that the correlation is significant at the 0.05 level (two-tailed); −, It indicate that the negative correlation.

However, in this experiment, when analyzing the Pearson correlation between the FC values before acupuncture in the sham-acupoint group and the neuropsychological scale scores, it was found that there was no correlation between the extracted FC values and the MMSE. Interestingly, a significant negative correlation was found between the FC values of Seed13-Seed7 and the MoCA (*r* = −0.563, *p* = 0.029). Moreover, in the results of the correlation analysis of the FC values before acupuncture in the ture-acupoint group, a negative correlation was also observed between the FC values of Seed13-Seed7 and the MoCA scores. See Table 10.

**Table 10:** Table of correlation analysis between FC values of seed points and other brain regions and neuropsychological scale scores in the sham - acupoint group before acupuncture.

Functional connectivity (FC)	Neuropsychological scale score	Pearson correlation	
		*r*	*P*
Seed7- Frontal_Mid_L	MMSE	0.147	0.601
Seed7- Frontal_Sup_Medial_L	MMSE	−0.057	0.840
Seed7- Precuneus_L	MMSE	0.133	0.637
Seed8- Frontal_Mid_L	MMSE	0.186	0.507
Seed8- Frontal_Sup_Medial_L	MMSE	−0.097	0.732
Seed8- Precuneus_L	MMSE	0.067	0.813
Seed13- Frontal_Mid_L	MMSE	−0.080	0.777
Seed13- Frontal_Sup_Medial_L	MMSE	−0.403	0.136
Seed13- Precuneus_L	MMSE	−0.088	0.755
Seed15- Frontal_Mid_L	MMSE	−0.017	0.951
Seed15- Frontal_Sup_Medial_L	MMSE	−0.120	0.670
Seed15- Precuneus_L	MMSE	−0.035	0.900
Seed17- Frontal_Mid_L	MMSE	−0.118	0.675
Seed17- Frontal_Sup_Medial_L	MMSE	−0.159	0.572
Seed17- Precuneus_L	MMSE	−0.056	0.843
Seed7- Frontal_Mid_L	MoCA	−0.232	0.405
Seed7- Frontal_Sup_Medial_L	MoCA	−0.165	0.557
Seed7- Precuneus_L	MoCA	−0.095	0.735
Seed8- Frontal_Mid_L	MoCA	−0.051	0.857
Seed8- Frontal_Sup_Medial_L	MoCA	−0.078	0.784
Seed8- Precuneus_L	MoCA	−0.325	0.237
Seed13- Frontal_Mid_L	MoCA	−0.034	0.903
Seed13- Frontal_Sup_Medial_L	MoCA	−0.563*	0.029
Seed13- Precuneus_L	MoCA	−0.482	0.069
Seed15- Frontal_Mid_L	MoCA	−0.029	0.918
Seed15- Frontal_Sup_Medial_L	MoCA	−0.418	0.121
Seed15- Precuneus_L	MoCA	−0.279	0.313
Seed17- Frontal_Mid_L	MoCA	−0.021	0.941
Seed17- Frontal_Sup_Medial_L	MoCA	−0.358	0.191
Seed17- Precuneus_L	MoCA	−0.270	0.330

*, It indicates that the correlation is significant at the 0.05 level (two-tailed); −, It indicate that the negative correlation.

**Figure 12:**
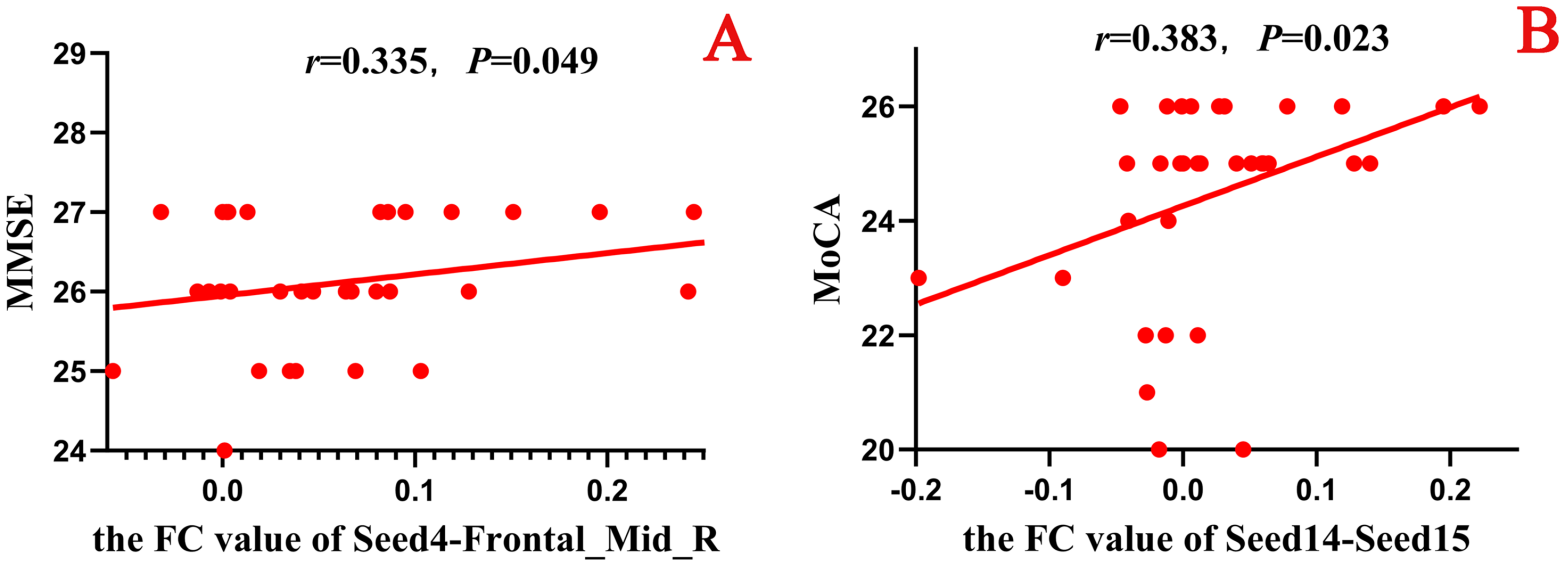
Scatter plot of the correlation analysis between the FC values between the seed points and brain regions after acupuncture and the scores of the neuropsychological scale in the true-acupoint group. *p* < 0.05 indicates statistical significance, r represents the correlation coefficient, and the red scatter plot indicates a positive correlation. Seed4: Frontal_Mid_Orb_R; Seed14: Cingulum_Ant_R; Seed15: Cingulum_Mid_L. **A** is a positive correlation analysis diagram of the FC values between seed4 - Frontal_Mid_R and the MMSE; **B** is a positive correlation analysis diagram of the FC values between seed14 - Seed15 and the MoCA.

**Figure 13:**
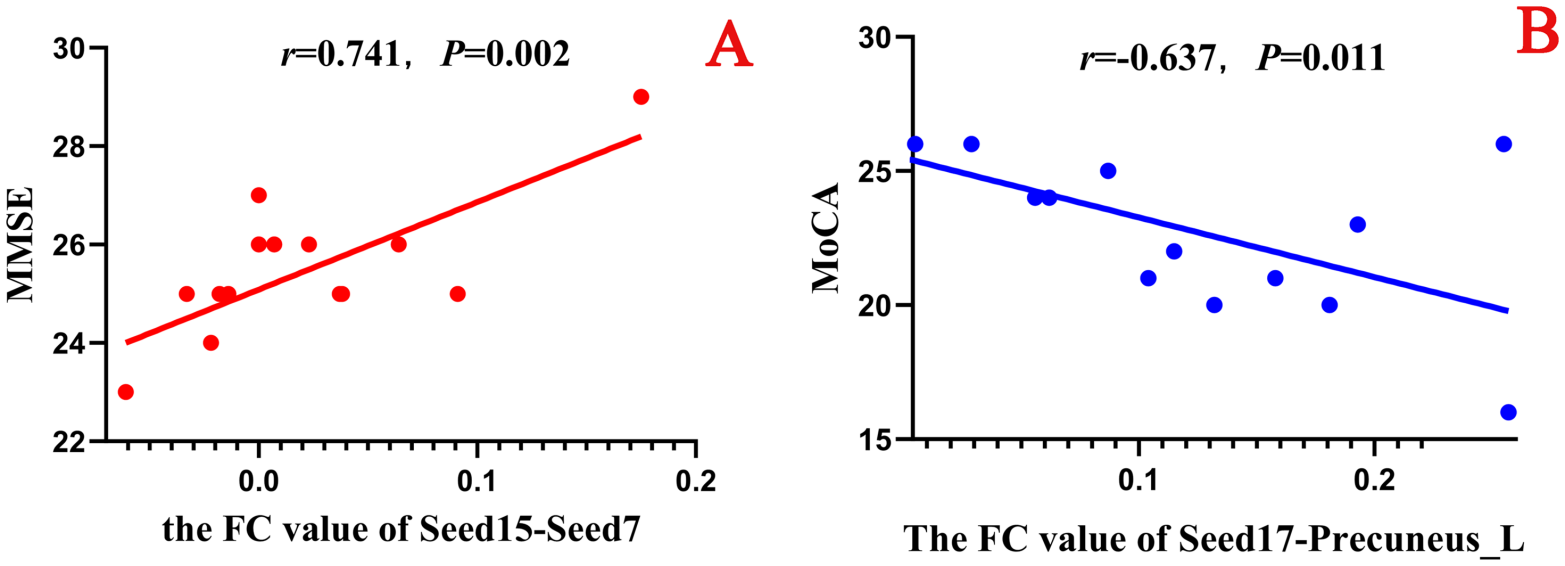
Scatter plot of the correlation analysis between the FC values between the seed points and brain regions after acupuncture and the scores of the neuropsychological scale in the sham-acupoint group. *p* < 0.05 indicates statistical significance, *r* represents the correlation coefficient, “-” indicates a negative correlation, the red scatter plot indicates a positive correlation, and the blue scatter plot indicates a negative correlation. Seed7: Frontal_Sup_Medial_L; Seed15: Cingulum_Mid_L; Seed17: Cingulum_Post_L. **A** is a positive correlation analysis diagram of the FC values between seed15 - Seed7 and the MMSE; **B** is a negative correlation analysis diagram of the FC values between seed17 - Precuneus_L and the MoCA.

In this study, a correlation analysis was further conducted between the FC values after acupuncture and the scores of the neuropsychological scale. The results showed that in both the true-acupoint group and the sham-acupoint group, there were few indicators showing a significant correlation between the FC values and the scale scores. In addition, the correlation analysis results between the FC values after acupuncture and the scores of the neuropsychological scale did not significantly overlap with those of the correlation analysis before acupuncture. The specific results are shown in Figures 12, 13.

## 4 Discussion

In traditional Chinese medicine (TCM) theory, aMCI falls within the categories of “forgetfulness” and “dementia.” It is believed that aMCI is caused by the decline of the internal organs and the deficiency of qi, blood, yin and yang, which leads to the brain lacking proper nourishment, and the location of the disease is in the brain (41). In Western medicine theory, Petersen et al. (42) defined MCI as a clinical and neuropsychological syndrome, characterized by cognitive impairment, and it is an intermediate state between physiological aging and dementia. Some studies have found that compared with naMCI, the abnormalities in the brain of aMCI patients are more severe, mainly manifested as memory loss. Its unique feature is the abnormal deposition of amyloid proteins, which is closely related to the progression of the disease in AD (43, 44).

Among the existing treatment methods, acupuncture therapy has emerged as a new approach. By stimulating specific acupoints, it can regulate the circulation of qi and blood in the human body as well as the functions of the internal organs. It can regulate multiple mechanisms as a whole to exert a neuroprotective effect, thereby improving the cognitive function status of patients. It has the advantages of minimal side effects, high safety, and personalized treatment. In the study by Bao et al. (45), from multiple aspects such as clinical symptoms, brain function, gut microbiota, and the expression of inflammatory cytokines, it was found that acupuncture therapy can improve the clinical symptoms of patients with aMCI. In addition, Wang et al. (46) discovered that acupuncture at the Taichong (LR3) and Hegu (LI4) acupoints can activate certain cognitive-related areas in patients with AD and MCI, mainly involving the temporal and frontal lobe regions, reflecting the specificity of the acupoints.

In this study, considering that not all patients with aMCI are suitable for treatment with the same acupoint, a group of acupoints was selected for intervention. At the same time, a sham acupoint group was also collected to rule out the brain effects caused by the pain of acupuncture, so as to accurately evaluate the effect of acupuncture at the acupoints.

In this study, we investigated the changes in functional connectivity within the limbic system of aMCI patients after acupuncture treatment based on fMRI. Firstly, on the premise of removing covariates such as age, gender, and years of education of the participants, it was found that compared with the HC group, there were significant differences in the FC between Seed14 of the true-acupoint group before acupuncture intervention and multiple brain regions. Specifically, the FC with Seed7 was significantly enhanced, and the FC with Seed15 was significantly weakened. At the same time, it was also found that the FC of Seed4, Seed29, and Seed30 in the sham-acupoint group before acupuncture intervention differed from that in the HC group. Through this comparison, the baseline differences in the FC between aMCI patients in different groups and healthy individuals before acupuncture were understood.

When performing the paired samples t-test within the groups of the true-acupoint group and the sham-acupoint group before and after acupuncture intervention, it was found that more changes in the FC between seed points and brain regions occurred in the true-acupoint group, mainly characterized by a decrease in FC. In contrast, most of the data in the sham-acupoint group did not pass the FEW correction, and there were no significant changes in the FC. This result suggests that the brain effects produced by acupuncture at true-acupoints are indeed more complex than those produced by acupuncture at sham-acupoints.

Secondly, we found that compared with the HC group, the changes in the FC of the seed points in both the true-acupoint group and the sham-acupoint group after acupuncture were mainly characterized by a decrease. The changes in the FC of the seed points in the true-acupoint group were more abundant, such as Seed3, Seed4, Seed7, Seed8, Seed13, Seed14, Seed15, Seed16, Seed17, Seed18, Seed25, and Seed29. While the changes in the FC of Seed7, Seed8, Seed13, Seed15, and Seed17 in the sham-acupoint group were relatively rich. The results showed that after acupuncture intervention, the left middle frontal gyrus ranked first in terms of both the frequency and the number of voxels of the FC changes between the seed points and this region, regardless of whether it was the true-acupoint group or the sham-acupoint group, and the number in the true-acupoint group was more than that in the sham-acupoint group. The right middle frontal gyrus became the specific brain effect mechanism of the intervention method in the true-acupoint group. As an important brain region of the prefrontal lobe, the middle frontal gyrus is responsible for the acquisition of human brain memory, learning, and stress awareness, and it completes the functional integration process of thinking and emotions. The weakening of the FC between this brain region and the seed points in the limbic system implies that acupuncture intervention will down-regulate the synchronous activity of neurons from different network regions of the brain.

Similarly, the changes in the FC between many seed points and the left superior frontal gyrus internalis (Seed23), as well as the left middle cingulate gyrus and the gyri surrounding its lateral side (Seed15), were also significantly weakened. Additionally, the decrease in the FC with the gyri below the bilateral parietal bones excluding the supramarginal gyrus and angular gyrus, as well as the precuneus, was also quite obvious. Most of these brain regions are located in the frontal and parietal lobes of the human brain, which are areas where the limbic system is distributed and where functional connectivity exists. These regions are involved in a variety of high-level functions of the human brain, such as emotional regulation, social cognition, executive control, and cognitive control.

Subsequently, when we compared the FC between Seed14 of the true acupoint group and the left superior frontal gyrus medialis (Seed7) as well as the right caudate nucleus with that of the HC group in two separate inter-group comparisons (before and after acupuncture), it was found that the FC was initially in an enhanced state, but after acupuncture, it showed a decreasing trend. These two changes in FC may potentially serve as observational indicators for the intervention of aMCI by acupuncture at acupoints. Regrettably, the FC values of Seed14-Seed7 and Seed14-right caudate nucleus extracted from the aMCI patients in the true-acupoint group did not show any correlation with the scores of neuropsychological scales. In the part of Pearson correlation analysis, it was found that the FC between multiple brain regions in the true-acupoint group before acupuncture was correlated with the MMSE and the MoCA. Among them, the FC values of Seed16-Seed15 and Seed13-Parietal_Inf_L were significantly negatively correlated with both MMSE and MoCA. In addition, the study also found that the extracted FC values were more often correlated with the MoCA scores, and the correlations were mainly negative. This implies that the MoCA scores are more representative in characterizing the abnormal functional connectivity between brain regions in aMCI patients. In the sham-acupoint group, the FC values extracted before acupuncture had no correlation with MMSE. Only the FC value of Seed13-Seed7 had a significant negative correlation with MoCA, and this result was also found in the correlation analysis of the true-acupoint group before acupuncture. It is well known that the middle frontal gyrus, as a crucial region of the prefrontal cortex, is responsible for memory learning and the acquisition of stress awareness, completing the functional integration of thought and emotion. However, the prefrontal cortex plays a vital role in various cognitive functions, including episodic memory, executive control, and reasoning abilities (47).

### 4.1 Limitations and implications of this study

This study was limited by research funding and time, resulting in a sample size that was not large enough, and only a small-sample experiment was completed. This may lead to the research results lacking sufficient representativeness and statistical power, affecting the generalizability of the conclusions. In addition, the functional connectivity analysis method based on seed points has certain limitations. This method is highly dependent on the selection of seed points. Once the seed region changes, the results of the functional connectivity analysis will change significantly accordingly. This means that the analysis process is extremely vulnerable to the interference of subjective factors of the researchers. Moreover, this analysis method is relatively conventional. When facing complex brain function research, it may not be able to fully explore the potential information in the data, and it is difficult to meet the current needs of in-depth research on brain functional connectivity.

## 5 Conclusion

During the experiment, abnormal functional connections were also observed between the limbic system and multiple sub-regions of the cerebellum. Previous studies have indicated that the cerebellum not only participates in motor regulation but also plays a significant role in memory and cognitive functions, and its abnormal activities may lead to impairments in executive functions and speech abilities (48). It is noteworthy that there is a limbic network in the cerebellum, which is unaffected by pathological changes during the prodromal stage of AD. In the stage of aMCI, the cerebellar limbic network can regulate the social cognitive function of patients through the mechanism of functional connectivity compensation (49).

In conclusion, this study indicates that the brain effect mechanism of acupuncture at acupoints in patients with aMCI is more complex. It can not only stimulate the functional connectivity between seed points within the limbic system, but also regulate the functional connectivity with other brain regions, mainly in a decreasing manner. Among them, the FC among Seed17-Parietal_Inf_L, Seed25-Frontal_Mid_L, and See25-Frontal_Sup_Medial_L has become a statistically significant detection index between the true-acupoint group of aMCI patients before and after acupuncture and the HC group.

## Data Availability

The raw data supporting the conclusions of this article will be made available by the authors, without undue reservation.
